# Interplay between N6-methyladenosine RNA methylation and protein lactylation: a novel crosstalk linking metabolism and epigenetic regulation in human diseases

**DOI:** 10.1186/s12967-026-08663-4

**Published:** 2026-07-20

**Authors:** Yanyan Gong, Yu Liu

**Affiliations:** School of Kinesiology and Health, Shenyang Sport University, Shenyang, 110102 China

**Keywords:** m6A, Protein lactylation, Metabolism, Epigenetics

## Abstract

N6-methyladenosine (m6A) RNA methylation is the most prevalent internal modification in eukaryotic mRNA and precisely regulates gene expression by regulating the RNA life cycle. Lactate, a central glycolytic metabolite, can transduce cellular metabolic states into epigenetic signals through protein lactylation, an emerging post-translational modification. Because both modifications are highly responsive to cellular metabolic status, they have emerged as important regulators of the metabolic-epigenetic interface. Increasing evidence supports a bidirectional regulatory crosstalk between m6A modification and protein lactylation. Lactate accumulation can modulate the expression and activity of m6A-related regulatory enzymes through histone and non-histone lactylation; conversely, m6A modification can reshape glycolysis and lactate metabolism, thereby altering lactate availability and protein lactylation. This reciprocal regulation has been implicated in a broad spectrum of diseases, including cancer, metabolic disorders, cardiovascular diseases, and immune-inflammatory conditions. In this review, the molecular mechanisms of m6A modification and protein lactylation are systematically summarized, with particular emphasis on their modes of interaction and pathological relevance. Current limitations and future perspectives are also discussed, providing a conceptual framework for elucidating disease mechanisms and developing therapeutic strategies targeting this regulatory network.

N6-methyladenosine (m6A) is the most abundant internal modification in eukaryotic mRNA and has attracted considerable attention in recent years. m6A dynamically regulates RNA fate through a coordinated system of “writers, erasers, and readers,” thereby affecting processes such as splicing, stability, translation, and degradation [1]. Research has shown that m6A is involved not only in cell differentiation, development, and immune regulation but also in pathological states, including tumor progression, metabolic disorders, and inflammation [2]. In addition, m6A modification is highly dependent on metabolic intermediates (e.g., SAM, α-KG), positioning it as a critical hub linking metabolism and post-transcriptional regulation [1]. Meanwhile, lactate, traditionally considered the end product of glycolysis, has been redefined as a key signaling molecule. In addition to mediating signal transduction via G protein-coupled receptors, lactate can serve as a substrate for protein lactylation, thereby modulating chromatin structure and protein function and ultimately shaping transcriptional regulation [3, 4]. Notably, both m6A RNA methylation and protein lactylation are representative metabolism-dependent epigenetic regulatory processes. Under conditions of metabolic reprogramming, these modifications do not act independently; instead, they form a multilayered and dynamically interactive regulatory network. On the one hand, lactate accumulation can regulate the transcription and functional activity of m6A-related enzymes such as METTL3 and ALKBH5 by inducing histone and non-histone lactylation, thereby modulating m6A levels [4, 5]. On the other hand, m6A can influence lactate production and lactylation levels by regulating glycolytic enzymes, lactate metabolic pathways, and hypoxia signaling such as HIF-1α [6, 7]. This bidirectional regulation not only enables metabolic states to be transmitted to epigenetic modifications but also feeds epigenetic regulation back into metabolic networks, potentially forming a multilayered closed-loop regulatory system. Epigenetic regulation serves as a critical bridge between genomic information and environmental changes. Recent studies have revealed that metabolic states not only provide energy and biosynthetic substrates but also modulate gene expression through epigenetic modifications. In complex pathological conditions, including cancer, metabolic diseases, and inflammation, metabolic reprogramming has been established as a central driver of disease initiation and progression [1, 3]. This review aims to systematically elucidate the fundamental molecular mechanisms of m6A and lactylation, with particular emphasis on their interaction patterns and roles in disease contexts, thereby providing a theoretical basis for understanding metabolism-driven disease mechanisms and guiding the development of potential therapeutic strategies.

Recently, the interplay among lactate metabolism, protein lactylation, and m6A RNA methylation has gained significant attention. However, current reviews mostly focus on cancer or single-disease perspectives, lacking a systematic, cross-disease overview. This review expands the research scope to diverse human conditions, including hematological malignancies, metabolic, cardiovascular, immune-inflammatory, fibrotic, and neurological diseases. We provide a comprehensive perspective on the common regulatory patterns and disease-specific variations within the m6A-lactylation axis. Mechanistically, we establish a three-layer regulatory framework. This framework clarifies the bidirectional relationship where lactylation modulates m6A modification, and m6A conversely regulates lactate homeostasis and lactylation through key signaling pathways. We also classify different types of evidence and highlight unresolved scientific questions in this field. Additionally, we summarize potential translational therapeutic strategies, focusing on targeting relevant molecules and implementing multimodal combination interventions.

We searched relevant literature from 2009 to 2026 using PubMed as the primary database. Other databases provided supplementary verification. Search terms encompassed m6A modification, lactate metabolism, protein lactylation, key molecules, and various human diseases. We included English original research and review articles with mechanistic or clinical evidence, while low-quality studies were excluded. As this is a narrative review, we did not perform the formal PRISMA systematic evaluation process.

## m6A RNA methylation

### Overview of m6A RNA methylation

As the most prevalent internal modification in eukaryotic mRNA, m6A represents a dynamic and reversible epitranscriptomic regulatory modality [8]. By coordinating RNA splicing, export, translation, and degradation, m6A constructs a sophisticated and dynamic gene expression regulatory network [9]. It influences gene expression across multiple stages of mRNA metabolism, including pre-mRNA processing, nuclear export, degradation, and translation, thereby playing essential roles in various physiological and pathophysiological processes [10]. As a key hub linking post-transcriptional regulation, metabolic status, and epigenetics, m6A provides new theoretical foundations for mechanistic studies of disease and the development of therapeutic targets [11].

The functional roles of m6A regulators in RNA metabolism are categorized into three types: “writers,” “erasers,” and “readers.” “Writers” mainly include METTL3, METTL14, METTL16, WTAP, KIAA1429, and RBM15. “Erasers” mainly include FTO and ALKBH5. “Readers” include nuclear readers, such as YTHDC1 and HNRNPA2B1, and cytoplasmic readers, such as YTHDF1-3, YTHDC2, and IGF2BP family proteins [8]. Beyond the conventional classification of m6A regulators as writers, erasers, and readers, their biological functions are also shaped by subcellular localization and context-dependent redistribution. For example, YTHDC1 is mainly enriched in nuclear speckles, where it participates in alternative splicing, nuclear RNA processing, and RNA export. It can also associate with m6A-modified lncRNAs such as XIST and MALAT1, whereas NEAT1-related paraspeckles may contribute to m6A-associated nuclear compartmental regulation. METTL3 and METTL14 are predominantly nuclear m6A writer components, but they may be recruited to DNA damage sites under genotoxic stress. In the cytoplasm, YTHDF1, YTHDF2, and YTHDF3 regulate translation, RNA decay, and RNA stability and can relocalize to stress granules during cellular stress. These findings suggest that m6A regulation is spatially compartmentalized rather than uniformly distributed, providing an additional layer of context-dependent control over RNA metabolism [8, 10, 12–15]. Extensive evidence indicates that m6A, by regulating RNA fate, m6A is broadly involved in stem cell self-renewal and differentiation and exerts diverse biological functions in cellular metabolism, organismal development, immune cell development, and immune responses. Mechanistically, m6A modification is dynamically orchestrated by writers, erasers, and readers to precisely regulate gene expression throughout the RNA life cycle, thereby forming the core of the epitranscriptomic regulatory network and providing a crucial mechanistic framework for the interface between RNA regulation and cellular metabolic status [10, 16]. The high sensitivity of m6A to cellular metabolic states suggests that it may act synergistically with other metabolism-dependent epigenetic mechanisms, laying the groundwork for investigating the m6A regulatory network from a metabolic perspective [17]. In addition to their molecular classification as writers, erasers, and readers, the biological functions of m6A regulators are strongly influenced by their subcellular localization. Different m6A regulators are enriched in distinct nuclear or cytoplasmic compartments, where specific steps of RNA metabolism, including pre-mRNA processing, nuclear export, translation, and RNA decay, are regulated. Therefore, understanding the spatial distribution of m6A-associated proteins is essential for interpreting their context-dependent functions in metabolism and disease.

**Table 1:** Subcellular localization and functional compartmentalization of major m6A regulators

Protein category	Representative proteins	Core localization		Specific sub-localization / notes		Major functions	Ref.
writers	METTL3, METTL14, WTAP, VIRMA/KIAA1429, RBM15, METTL16		Nucleus		Enriched in nuclear speckles; METTL3/METTL14 may be recruited to DNA damage sites under genotoxic stress	m6A deposition, pre-mRNA processing, and RNA fate regulation	[8–10, 12, 13]
erasers	FTO, ALKBH5		Nucleus and/or cytoplasm		ALKBH5 is mainly nuclear; FTO may partially shuttle between the nucleus and cytoplasm in a context-dependent manner	demethylation and regulation of RNA stability and metabolism	[8–10, 12]
Nuclear readers	YTHDC1, HNRNPA2B1		Nucleus		Enriched in nuclear speckles; associated with m6A-modified lncRNAs such as XIST and MALAT1; NEAT1-related paraspeckles may contribute to nuclear compartmental regulation.	Alternative splicing, nuclear RNA processing, and RNA export	[8–10, 12, 14]
Cytoplasmic readers	YTHDF1, YTHDF2, YTHDF3, YTHDC2, IGF2BP family		Cytoplasm		YTHDF proteins may relocalize to stress granules under cellular stress	Translation regulation, RNA degradation, RNA stabilization, and stress responses	[8–10, 12, 15]

**Notes**: The subcellular localizations listed in this table reflect predominant or context-dependent distributions of major m6A regulators reported in the literature. These regulators may undergo dynamic relocalization in response to cellular stress, DNA damage, metabolic remodeling, or lncRNA-mediated nuclear compartmentalization. Thus, the indicated compartments should be regarded as functional localization patterns rather than invariant locations across all cell types, disease states, or experimental conditions

Notably, the localization of m6A regulators is dynamic rather than fixed. YTHDC1 is mainly enriched in nuclear speckles but may redistribute to paraspeckles through interactions with specific lncRNAs such as XIST or NEAT1. METTL3 and METTL14 may be recruited to DNA double-strand break sites, whereas YTHDF1–3 can participate in stress granule assembly under cellular stress. These dynamic localization patterns suggest that m6A-mediated regulation is spatially compartmentalized and may vary across cell types, metabolic states, and disease contexts.

### Metabolic regulation of m6A

**Fig. 1:**
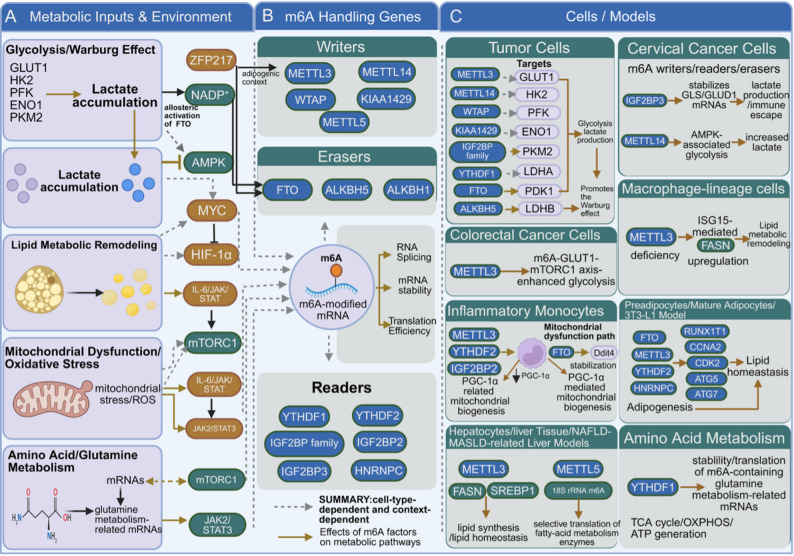
Metabolic regulation of m6A machinery across distinct cellular contexts. (**A**) Metabolic inputs and environmental stress activate downstream signaling pathways to modulate m6A modifications. (**B**) The m6A machinery consists of writers, erasers, and readers. These factors dictate the post-transcriptional fate of metabolism-related transcripts by regulating RNA splicing, mRNA stability, and translation efficiency. (**C**) The metabolic impact of the m6A machinery is highly cell-type and context-dependent. In tumor cells, m6A factors promote glycolysis and the Warburg effect. In immune and metabolic models, they regulate lipid remodeling, mitochondrial function, adipogenesis, and amino acid metabolism. Dashed arrows indicate upstream metabolic regulation of m6A factors. Solid arrows indicate downstream effects on metabolic pathways

m6A not only regulates cellular metabolic states but also contributes to the regulation of multiple key metabolic pathways by controlling the post-transcriptional fate of metabolism-related genes. At the post-transcriptional level, dynamic and reversible m6A modifications regulate RNA splicing, translation efficiency, and stability, thereby contributing extensively to diverse physiological and pathological processes [18]. Recent studies have revealed that m6A exerts prominent regulatory effects and plays essential roles in glycolysis, lactate production, lipid metabolism, mitochondrial function, and amino acid metabolism, demonstrating significant biological and pathological implications in the onset and progression of related diseases [19, 20] (Fig. 1).

Glycolysis is the process by which one glucose molecule is converted into two pyruvate molecules, yielding two molecules of adenosine triphosphate (ATP) [21]. As the central pathway of glucose metabolism, glycolysis is regulated by m6A modification, which serves as a vital mechanism for glucose homeostasis and downstream effects [19]. On one hand, m6A writers, including METTL3, METTL14, WTAP, and KIAA1429, and reader proteins, such as the IGF2BP family and YTHDF1, enhance glucose uptake and glycolytic flux by increasing the mRNA stability or translation efficiency of glucose transporters such as GLUT1 and key glycolytic enzymes such as HK2, PFK, ENO1, and PKM2, thereby promoting the generation of ATP and metabolic intermediates. On the other hand, m6A modification directs carbon flux from mitochondrial oxidative phosphorylation (OXPHOS) toward the glycolytic pathway by regulating key nodes of pyruvate metabolism, such as PDK4 and LDHB. Meanwhile, m6A demethylases, including FTO and ALKBH5, can activate lncRNAs or autophagy-related genes by modulating signaling pathways and can act synergistically with MYC, HIF-1α, and IL-6/JAK/STAT pathways to further enhance glycolytic capacity [18]. Because tumor cells typically exhibit markedly enhanced aerobic glycolysis, known as the Warburg effect, cancer has become the most representative and extensively studied pathological model for investigating m6A-mediated glycolytic reprogramming [7, 22, 23]. Existing literature indicates that m6A modification regulates tumor cell glycolysis and proliferation in multiple cancer types, including pancreatic cancer, colorectal cancer, cervical cancer, and triple-negative breast cancer; for instance, m6A can directly modify the mRNA of key glycolytic enzymes or glycolysis-related factors, such as HK2, GLUT1, ENO1, PDK4, and LDHA, thereby regulating tumor glycolysis by influencing their expression [6, 7, 24–26].

Lactate is the major product of glycolysis, and enhanced glycolysis leads to substantial lactate accumulation, a process in which m6A plays a critical role [1]. m6A may promote the conversion of pyruvate to lactate by regulating the fate of transcripts encoding lactate dehydrogenases, such as LDHA, and monocarboxylate transporters, including MCT1 and MCT4. First, m6A can directly modify and stabilize the mRNA of key glycolysis-related factors, including HK2, GLUT1, ENO1, and PDK4, thereby promoting glucose uptake and enhancing glycolytic flux [7]. Second, m6A acts on critical nodes in pyruvate metabolism, such as LDHA and pyruvate dehydrogenase kinase 1 (PDK1), thereby influencing whether pyruvate is directed toward lactate production or mitochondrial oxidative metabolism. Furthermore, m6A is extensively involved in the initiation and progression of various diseases, including cancer, metabolic disorders, and inflammation, by regulating glycolysis and lactate-related metabolic pathways. For example, IGF2BP3 stabilizes the mRNA of GLS and glutamate dehydrogenase 1 (GLUD1) via m6A modification, thereby promoting lactate production and mediating immune escape in cervical cancer cells [1, 27–31]. Studies have also shown that METTL14 increases lactate levels through AMPK by regulating glycolytic pathways, thereby promoting cervical cancer progression [24] (Fig. 1). These findings demonstrate that m6A exerts a fundamental role in metabolic regulation during disease progression by regulating glycolysis and lactate-related metabolic states.

Lipid metabolism is a complex physiological process involving nutrient, hormonal, and homeostatic regulation, as well as multiple molecular factors and signaling pathways critical for human growth and development [20]. Recent studies indicate that, in metabolism-related cells, tissues, and experimental models, including adipose tissue, preadipocyte and mature adipocyte models such as 3T3-L1 adipogenesis models, hepatocytes, liver tissue, and NAFLD/MASLD-related liver models, m6A RNA methylation plays a vital role in lipid metabolism by regulating the stability, translation efficiency, and splicing of key lipid metabolism-related genes, thereby influencing adipogenesis, lipid synthesis, hepatic steatosis, and metabolic homeostasis [9, 18, 20, 32–34]. As a major m6A demethylase, FTO is associated with increased production of pro-adipogenic short isoforms and drives mitotic clonal expansion (MCE) by regulating the alternative splicing of the adipogenic regulator RUNX1T1. Concurrently, FTO supports cell cycle-related genes, including CCNA2 and CDK2, and preventing YTHDF2-mediated mRNA degradation. Additionally, FTO maintains the autophagic activity required to promote adipocyte maturation by regulating the m6A levels of autophagy-related genes such as ATG5 and ATG7. In contrast, METTL3-mediated m6A writing often exhibits inhibitory effects, with strong dependence on cell type and context. At the level of lipid synthesis, m6A precisely regulates lipid synthesis flux by directly regulating the mRNA stability and translation of key enzymes, such as FASN and SREBP1, and through lncRNA-mediated chromatin and transcriptional regulation. In terms of overall lipid homeostasis, m6A modification may help maintain a dynamic balance between lipid synthesis and degradation by integrating metabolic states, signaling pathways, and translational regulation. Various m6A reader proteins, such as HNRNPC and IGF2BP3, influence cytoskeletal remodeling, signal transduction, including the ERK and JAK-STAT pathways, and adipogenic decisions by stabilizing or inhibiting specific transcripts. Upstream metabolites and transcription factors further regulate m6A enzyme activity; for example, NADP⁺ serves as an allosteric activator of FTO, directly enhancing its demethylation activity and feeding back cellular metabolic states to the epitranscriptomic level. The transcription factor ZFP217 amplifies or inhibits m6A dependent adipogenic signaling by synergistically regulating the expression and function of FTO and METTL3. Furthermore, beyond mRNA, rRNA m6A modifications such as METTL5-mediated 18S rRNA m6A, regulate the protein synthesis of key fatty acid metabolism enzymes by influencing ribosome assembly and selective translation, providing a new layer of lipid homeostasis regulation [18, 32]. Notably, the effects of m6A on lipid metabolism are highly site and context dependent, with distinct modification sites and reader interactions potentially leading to opposite metabolic outcomes. Dysregulation of these mechanisms is commonly observed in obesity, non-alcoholic fatty liver disease, and other metabolic disorders, underscoring the importance of m6A mediated lipid regulation in metabolic homeostasis and disease progression [20] (Fig. 1).

Notably, METTL3 exhibits context-dependent, divergent, and even opposing regulatory effects across distinct metabolic pathways and cellular compartments [7, 19]. In macrophage-associated lipid metabolism, METTL3 deficiency in myeloid or macrophage-lineage cells can promote tumor progression by facilitating ISG15-mediated FASN upregulation and lipid metabolic remodeling, thereby supporting a pro-tumorigenic macrophage phenotype [35]. Conversely, in glycolytic tumor-cell contexts, elevated METTL3 enhances glycolytic activity by stabilizing m6A-modified glycolysis-related transcripts or upstream regulators [7, 19, 36, 37]. For example, METTL3 activates the m6A-GLUT1-mTORC1 axis in colorectal cancer [37] (Fig. 1).

These findings suggest that the apparent paradox of METTL3 function largely depends on substrate specificity and cellular context. The functional outcome of METTL3 is dictated by the specific metabolic target transcripts being methylated and the metabolic state of the cell [7, 19, 35–37]. Therefore, METTL3 should not be regarded as uniformly oncogenic or uniformly tumor-suppressive across all contexts; rather, its role is jointly determined by cellular lineage, disease context, metabolic pathway, and the target mRNA landscape. This supports the view that METTL3-mediated m6A modification functions as a flexible rheostat in metabolic regulation rather than a rigid binary switch [7, 19].

During cellular metabolic reprogramming, m6A not only participates in the remodeling of energy supply by regulating glycolysis, lipid metabolism, and amino acid metabolism but also plays a key role in maintaining metabolic homeostasis and modulating disease progression by fine-tuning mitochondrial function, the core hub of cellular energy metabolism. m6A writers and readers, such as METTL3, YTHDF2, and IGF2BP2, can suppress mitochondrial biogenesis and ATP production by reducing the expression of master regulators such as PGC-1α, leading to impaired mitochondrial function, aggravated oxidative stress, and mitochondrial damage in inflammatory monocytes, thereby promoting inflammation-related metabolic diseases such as atherosclerosis. Conversely, the demethylase FTO enhances PGC-1α-mediated mitochondrial biogenesis and aerobic metabolism by stabilizing transcripts such as Ddit4 through reduced m6A levels, thereby exerting protective effects in metabolic syndrome, obesity, and lipid disorders. m6A also influences mitochondrial quality control and stress-induced apoptosis by regulating mitochondrial fission/fusion and signaling pathways such as mTORC1 and JAK2/STAT3, thereby participating in adipogenesis, muscle differentiation, and tumor energy metabolic reprogramming. Non-canonical RNA modifiers such as ALKBH1 can localize to mitochondria and influence cell proliferation, suggesting that m6A-related regulation may have more direct mitochondrial roles in tumorigenesis and stem cell homeostasis. In summary, m6A RNA modification plays a central role in metabolic remodeling across various diseases by regulating mitochondrial biogenesis, mitochondrial dynamics, and energy metabolism pathways [38]. In amino acid metabolism, although research remains relatively limited, evidence suggests that m6A affects the expression of amino acid transporters and key metabolic enzymes. For instance, the reader YTHDF1 recognizes m6A-containing glutamine metabolism-related mRNAs, promoting their stability and translation to enhance glutamine pathway activity [19, 39]. Recent studies have shown that m6A influences the TCA cycle, OXPHOS, and ATP generation by regulating key glutamine metabolism enzymes and mitochondrial genes, playing a central regulatory role in tumor metabolic reprogramming and various metabolic diseases [38, 40, 41] (Fig. 1). Collectively, m6A establishes a multi-pathway metabolic regulatory network through its extensive involvement in glycolysis, lipid metabolism, mitochondrial function, and amino acid metabolism, thereby providing a vital metabolic foundation for m6A-mediated pathological functions.

## Lactate and protein lactylation

### Lactate as a metabolic signal

Lactate exerts a wide range of biological functions, including the mediation of histone and non-histone lactylation, the regulation of immunosuppression, the activation of signal transduction pathways, and its function as an energy substrate. Historically, lactate was viewed as a metabolic waste product of glycolysis that was produced when ATP demand exceeded oxygen supply. However, with advances in research on metabolic reprogramming and immunometabolism, its biological functions have been redefined1. Lactate is now recognized not only as an energy substrate but also as a signaling molecule involved in the regulation of cellular metabolic activities [1, 42].

**Fig. 2:**
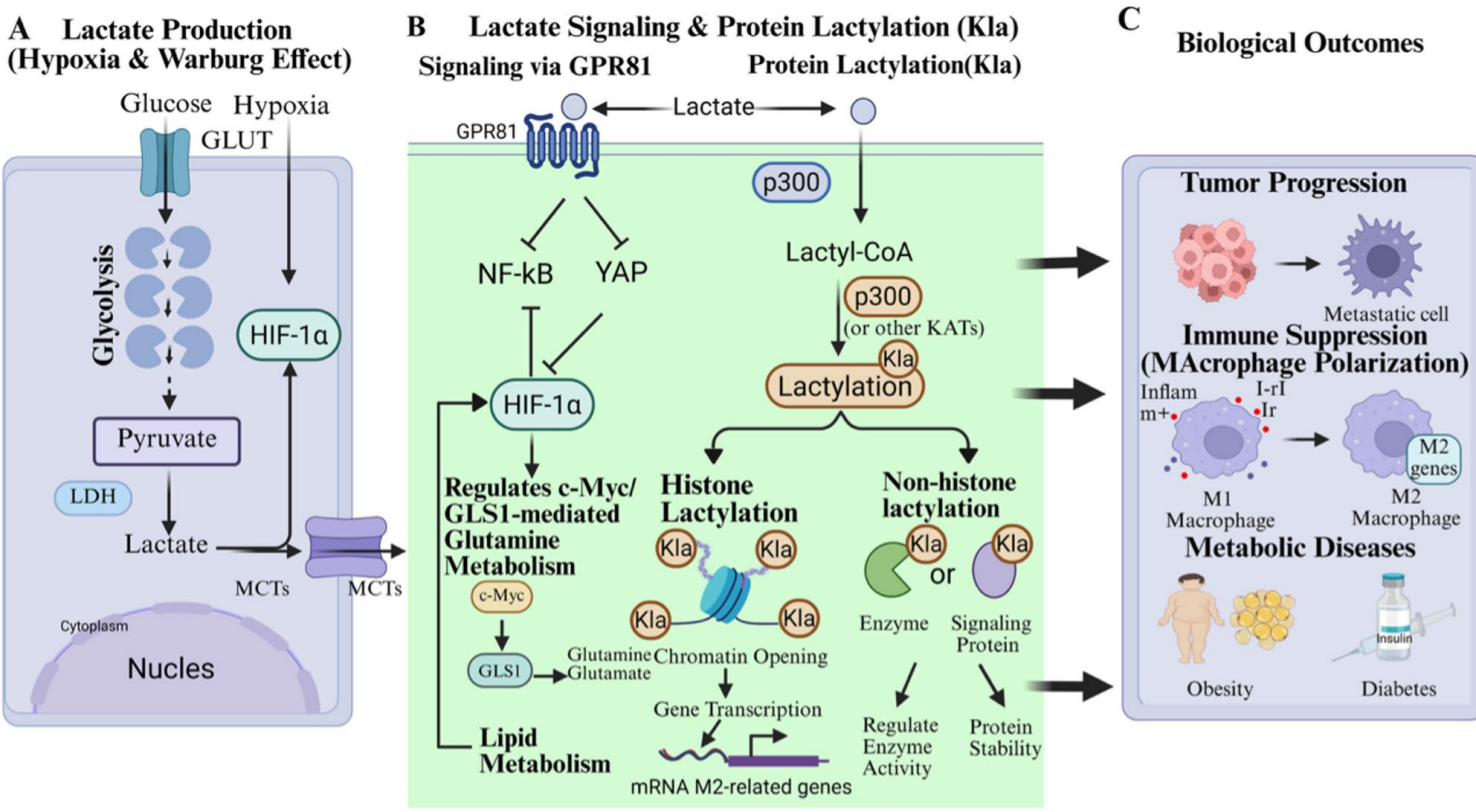
The molecular landscape of lactate production, signaling, and protein lactylation in health and disease. (**A**) Hypoxia and the Warburg effect increase glucose uptake and glycolysis. LDH converts pyruvate to lactate, which is transported by MCTs. (**B**) Lactate activates GPR81 signaling and regulates NF-κB, YAP, and HIF-1α. Lactyl-CoA supports p300 or KAT mediated protein lactylation. Histone lactylation promotes chromatin opening and gene transcription, whereas non-histone lactylation regulates enzyme activity and protein stability. (**C**) Lactate signaling and lactylation contribute to tumor progression, macrophage polarization, immune suppression, obesity, and diabetes

Lactate functions as a signaling molecule by acting as an agonist for the G protein-coupled receptor GPR81, thereby mediating various biological functions [5, 43]. For example, lactate can inhibit the activation of YAP-related proteins and NF-κB through GPR81-mediated signaling, thereby suppressing the pro-inflammatory response of macrophages to LPS stimulation [44]. Lactate also reprograms cellular energy metabolism via GPR81, thereby regulating the malignant phenotype of breast cancer. Lactate is associated with tumor growth and invasiveness by modulating extracellular matrix (ECM) properties and Notch ligand signaling [45, 46]. Additionally, lactate participates in other metabolic pathways, such as activating c-Myc to upregulate GLS1 expression, thereby enhancing glutamine uptake and metabolism in oxidative cancer cells [47]. It also serves as a regulator of fatty acid oxidation. Lactate accumulation can increase intracellular fatty acid synthesis while inhibiting β-oxidation [48]. Through its roles in metabolic regulation and signal transduction, lactate exerts broad regulatory effects in physiological and pathological processes. In tumors, lactate accumulation promotes metabolic reprogramming and immune escape, thereby contributing to drug resistance. Elevated lactate levels can induce phenotypic changes in macrophages, thereby regulating inflammatory responses [49]. As research progressed, lactylation was identified as a novel lactate-related post-translational modification [50] (Fig. 2).

### Protein lactylation

Protein lactylation is a recently identified post-translational modification derived from lactate, in which lysine residues are covalently modified by lactate-derived lactyl groups to form lysine lactylation (Kla) [51]. This modification was first systematically characterized in 2019 by Zhang et al., who demonstrated at the molecular level that lactate is not merely a glycolytic end product but can directly participate in chromatin regulation and gene expression. It was further demonstrated that lactate-derived lactyl modifications can stimulate gene transcription and directly affect transcriptional activity [4]. The occurrence of protein lactylation is highly dependent on intracellular glycolytic activity and the degree of lactate accumulation. Under conditions of hypoxia, enhanced glycolysis, or inflammatory stimulation, lactate production increases significantly and is accompanied by dynamic elevations in histone lactylation levels (Fig. 2). This evidence establishes protein lactylation as a metabolism-dependent epigenetic regulatory mechanism [4, 52].

Protein lactylation is a metabolism-dependent post-translational modification driven by glycolytic flux and lactate accumulation. When glycolytic flux is enhanced or intracellular lactate levels and LDH activity are increased, protein lactylation levels rise significantly. This supports the notion that lactate from glycolysis is a key driver of lactylation, and the modification is dependent on the glycolysis-lactate axis [53, 54]. Protein lactylation primarily targets histone and non-histone substrates. In the context of m6A–lactylation crosstalk, these lactylation events can be further discussed at three functional levels: histone lactylation-mediated transcriptional regulation, non-histone lactylation-mediated modulation of protein stability or activity, and direct lactylation of m6A regulatory enzymes that may influence RNA methylation dynamics. Histone lactylation participates in epigenetic regulation, whereas non-histone lactylation regulates protein function and cellular metabolism, together forming an integrated regulatory network. Current evidence suggests that histone lactylation is enriched at specific sites associated with transcriptionally active regions, whereas non-histone lactylation may influence biological functions by altering protein conformation, stability, or interaction patterns. Furthermore, lactylation often coexists with acetylation and butyrylation at lysine sites, suggesting that different metabolism-dependent PTMs may form combinatorial modification patterns on the same protein or site to achieve fine-tuned control of protein function [53, 55]. Lactylation participates in cellular metabolic regulation through multilevel mechanisms and is considered a key downstream effect of lactate signaling [4]. At the transcriptional level, histone lactylation can alter chromatin conformation and promote the transcription of genes related to glycolysis, lactate production, and inflammation, thereby facilitating a shift toward enhanced glycolysis and metabolic reprogramming [56]. At the enzyme regulation level, non-histone lactylation can directly act on key metabolic enzymes, affecting their catalytic activity or stability and thereby regulating the metabolic partition between glycolysis and the TCA cycle [53]. In metabolic-signaling integration, lactylation acts as a core component of the lactate signaling pathway, working with GPR81 and HIF-1α to form an integrated “metabolite-signaling-epigenetic” regulatory model [4, 43] (Fig. 2).

In recent years, accumulating evidence has demonstrated that protein lactylation is widely involved in metabolic dysregulation associated with various diseases. In tumors, the persistent Warburg effect leads to substantial lactate accumulation, thereby significantly enhancing lactylation levels. Histone lactylation promotes the expression of genes related to glycolysis and immunosuppression, thereby supporting tumor proliferation, survival, and immune escape while reshaping the metabolic and epigenetic landscape of the tumor microenvironment [52]. In inflammatory and immune diseases, the lactate-lactylation axis influences the intensity and outcome of inflammatory responses by regulating the metabolic state and functional phenotype of immune cells. For example, it participates in the transition of macrophages from a pro-inflammatory to a reparative or immunosuppressive phenotype [57]. In metabolic diseases such as obesity and diabetes, abnormal lactate accumulation and elevated lactylation levels may contribute to lipid metabolic imbalance and chronic low-grade inflammation, providing evidence for a new epigenetic mechanism of metabolic homeostatic disruption [58]. In summary, protein lactylation establishes a vital molecular bridge between metabolic reprogramming and cell fate regulation by directly converting lactate-associated metabolic states into alterations in protein function and gene expression (Fig. 2).

## Interplay between m6A and lactylation

m6A methylation and protein lactylation are not isolated epigenetic modifications; rather, they form an interconnected regulatory network in the context of metabolic reprogramming [17]. Lactate, as a metabolite, directly or indirectly regulates the expression and catalytic activity of m6A regulatory proteins through lactylation. Conversely, m6A influences lactate production and transport through metabolic reprogramming pathways, thereby indirectly affecting lactylation levels [1, 4]. Thus, a bidirectional regulatory network exists between m6A and lactylation [17].

### Lactylation-driven regulation of m6A

Lactate functions not merely as the terminal product of glycolysis but also as a metabolic source for protein lactylation, thereby converting cellular metabolic states into regulatory signals that influence chromatin remodeling, protein function, and RNA modification [1, 4, 51]. Based on available evidence, lactylation-mediated regulation of the m6A system can be conceptualized across three distinct layers: histone lactylation-mediated transcriptional regulation, non-histone lactylation-mediated modulation of protein function, and the direct lactylation of RNA-modifying enzymes or m6A regulatory proteins themselves [51, 53, 55, 59].

#### Histone lactylation-mediated transcriptional regulation of m6A regulators

At the chromatin level, lactate accumulation induces acyltransferase-dependent histone lactylation (associated with enzymes such as p300), particularly marks like H3K18la and H3K9la. These modifications are typically enriched within the promoter or enhancer regions of transcriptionally active genes, thereby modulating their transcriptional activity [4, 56, 60]. Within the context of the m6A system, histone lactylation has been associated with increased transcription of m6A regulators and may alter m6A landscapes in specific cellular or disease models [33, 61, 62].

**Fig. 3:**
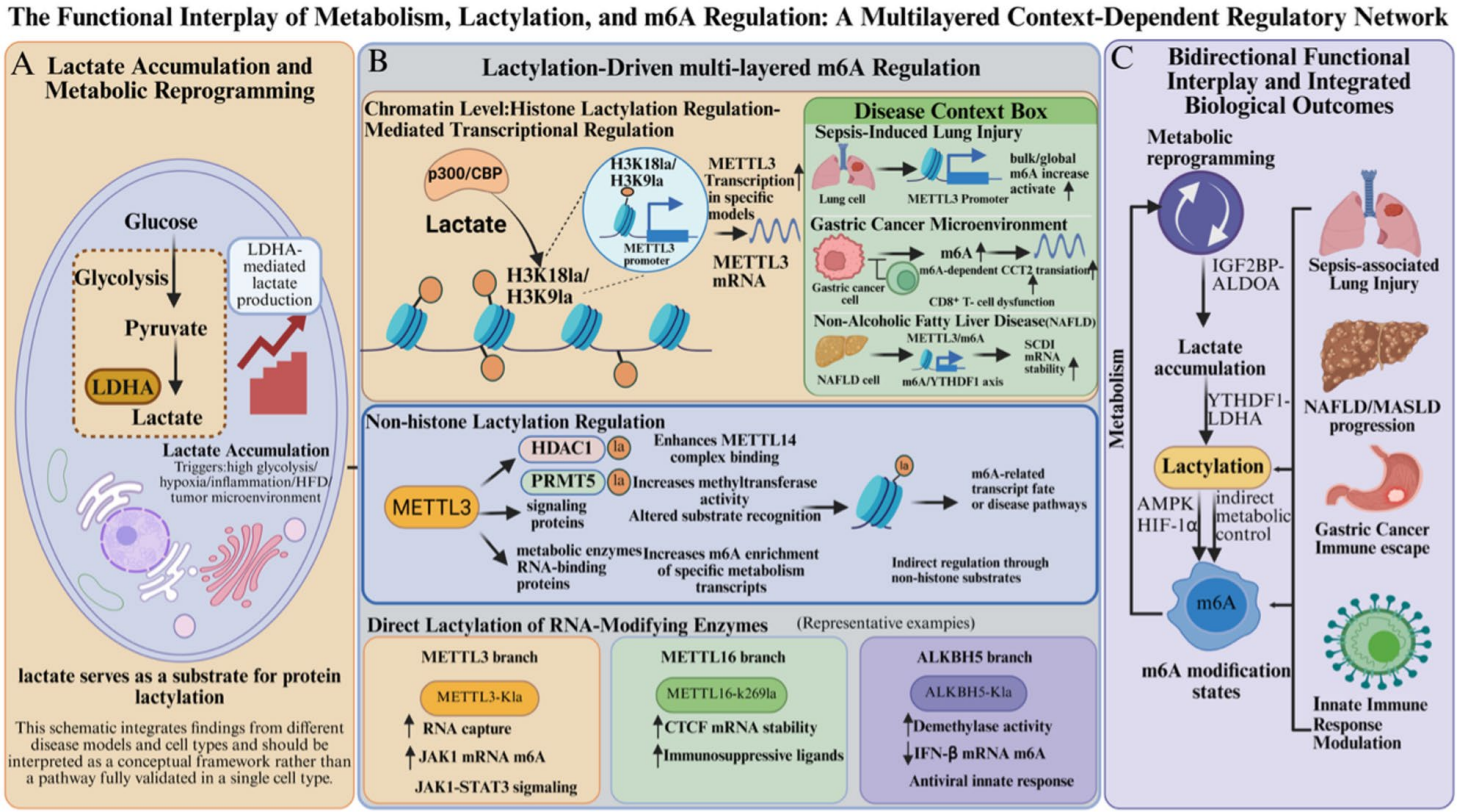
The functional interplay of metabolism, lactylation, and m6A regulation: a multilayered context-dependent regulatory network. (**A**) Hypoxia, inflammation, high-fat diet, and tumor microenvironmental stress enhance glycolysis and LDHA-mediated lactate production. Lactate accumulation provides the substrate for protein lactylation and links metabolic reprogramming to epigenetic regulation. (**B**) Lactylation regulates m6A at several levels. Histone lactylation, mainly H3K18la and H3K9la, can increase METTL3 transcription in sepsis-associated lung injury, gastric cancer, and NAFLD/MASLD models. Non-histone lactylation may alter the activity or interactions of HDAC1, PRMT5, metabolic enzymes, signaling proteins, and RNA-binding proteins. Direct lactylation of METTL3, METTL16, and ALKBH5 affects RNA binding, mRNA stability, demethylase activity, and downstream signaling. (**C**) m6A reciprocally controls glycolysis, lactate production, and lactylation through the IGF2BP2–ALDOA and YTHDF1–LDHA axes, as well as AMPK and HIF-1α signaling. This bidirectional network contributes to sepsis-associated lung injury, NAFLD/MASLD progression, gastric cancer immune escape, and innate immune regulation. The pathways shown were identified in different cell types and disease models and are presented as an integrated conceptual framework

For instance, in sepsis-associated lung injury models, LDHA-mediated lactate accumulation is linked to elevated H3K18la levels, where the enrichment of H3K18la at the METTL3 promoter region correlates with enhanced METTL3 transcription and a corresponding increase in global m6A levels within these models [61]. Similarly, within the gastric cancer microenvironment, lactate-induced H3K18la enrichment correlates with increased METTL3 expression, which in turn enhances CCT2 translation in an m6A-dependent manner, thereby contributing to CD8 + T cell dysfunction and tumor immune evasion [62]. Furthermore, in non-alcoholic fatty liver disease (NAFLD) models, a high-fat diet induces LDHA expression and lactate accumulation, promoting H3K18la enrichment at the METTL3 promoter region and subsequently modulating the stability of lipid metabolism-related transcripts, such as SCD1, via the METTL3/ m6A/YTHDF1 axis [33] (Fig. 3). However, this histone lactylation–m6A regulator axis has been validated mainly in specific experimental models, and its generalizability across different tissues, cell types, disease stages, and lactate-rich microenvironments remains to be further established.

Histone lactylation may indirectly affect disease phenotypes by altering the expression of m6A regulators involved in RNA processing and alternative splicing, although this regulatory axis remains insufficiently validated. As key components of the m6A recognition system, m6A readers broadly regulate RNA fate, including RNA processing, nuclear export, stability, translation, and degradation [8, 10, 12]. Nuclear readers such as YTHDC1 and HNRNPA2B1 participate in pre-mRNA processing and splice-site selection, suggesting that changes in their expression or activity could influence m6A-dependent isoform selection [63–65]. These alternatively spliced isoforms may differ in protein structure, stability, localization, and signaling capacity, thereby affecting cell survival, apoptosis resistance, migration, metastasis, and therapeutic response [66, 67]. From a functional perspective, alternative splicing isoforms generated by m6A reader-dependent regulation have been linked to three major biological dimensions in cancer. First, regarding cell survival, alternative splicing can produce pro-apoptotic versus anti-apoptotic isoforms of key regulators such as BCL2 family members, thereby modulating cellular sensitivity to metabolic stress and cytotoxic stimuli. Second, in tumor metastasis, splicing variants of transcription factors (e.g., ZEB1/2, Snail) or adhesion molecules can promote epithelial-mesenchymal transition (EMT) and invasive phenotypes, facilitating cancer cell dissemination. Third, for therapeutic resistance, alternative splicing represents a well-established adaptive mechanism by which cancer cells generate drug-resistant isoforms of target proteins (e.g., AR-V7 in prostate cancer), bypass apoptotic checkpoints, or upregulate DNA repair components (e.g., ERCC1) to evade treatment. Collectively, these observations suggest a conceptual framework in which lactate-enriched metabolic stress—acting through histone lactylation and subsequent upregulation of nuclear m6A readers—may potentiate the production of pro-survival, pro-metastatic, and drug-resistant mRNA isoforms. However, direct experimental validation of this complete cascade remains an important direction for future investigation. In tumors, such isoform switching may favor pro-survival, pro-invasive, or drug-resistant phenotypes; however, whether lactate-enriched metabolic stress directly regulates this process through histone lactylation-dependent control of m6A readers remains to be experimentally established [64, 66, 67]. Therefore, the proposed link among histone lactylation, m6A reader expression, alternative splicing, and disease progression should currently be interpreted as a plausible but incompletely validated mechanism requiring isoform-level, chromatin-level and rescue based studies.

#### Non-histone lactylation-mediated regulation of protein stability and function

Beyond histones, lactylation can also occur on various non-histone substrates, thereby influencing cellular functions by altering protein conformation, stability, subcellular localization, protein-protein interactions, RNA-binding capacity, or enzymatic activity [53, 55, 59]. Distinct from histone lactylation, which predominantly operates at the level of chromatin transcriptional regulation, non-histone lactylation highlights the direct modulation of the functional status of the target proteins themselves [51, 53, 59]. This layer differs from the direct lactylation of core RNA-modifying enzymes discussed below, because many non-histone lactylation events affect the m6A network indirectly through signaling proteins, metabolic enzymes, chromatin regulators, or RNA-binding proteins.

Within the m6A–lactylation crosstalk, non-histone lactylation can modulate the m6A regulatory network through two primary modalities. The first modality involves lactylation targeting regulatory proteins outside the canonical m6A pathway—such as HDAC1 and PRMT5—thereby influencing ferroptosis-associated pathways, therapeutic resistance phenotypes, or indirectly altering m6A-related post-transcriptional regulatory processes [68, 69]. The second modality features the direct lactylation of m6A regulatory components or RNA-binding proteins—such as VIRMA, YTHDF3, or IGF2BP3—subsequently affecting the recognition, stability, or translational efficiency of m6A-modified transcripts [70–73] (Fig. 3). Nevertheless, many reported non-histone lactylation events are linked to m6A regulation through pathway-level or functional associations rather than direct biochemical evidence, and therefore should be interpreted according to the specific substrate, cell type, and experimental system.

#### Direct lactylation of RNA-modifying enzymes

The third mechanistic layer involves the direct lactylation of RNA-modifying enzymes or m6A regulatory proteins themselves, which directly impacts m6A writing, erasing, or recognition processes [74–76]. Compared with the former two mechanisms, this tier of regulation is more intrinsic to the m6A machinery itself, thereby holding more profound mechanistic significance when supported by robust evidence.

Existing studies indicate that under hyperlactic or highly glycolytic conditions, METTL3 can undergo lysine lactylation [74]. This lactylation event has been reported to enhance the RNA-capturing capacity of METTL3 and promotes the m6A modification of JAK1 mRNA, subsequently modulating downstream JAK1–STAT3 signaling cascades [74].

Furthermore, in pancreatic ductal adenocarcinoma-associated tumor-bound Schwann cell models, METTL16 lactylation at K269 has been reported to augment the m6A-dependent stability of CTCF mRNA and activate the expression of immunosuppressive ligands [76] (Fig. 3). However, this specific mechanism should be strictly contextualized within particular cell types and disease settings, rather than generalized as a universal phenomenon for all METTL16-dependent m6A regulation. Similarly, in the context of viral infection or innate immunity, ALKBH5 lactylation facilitates its demethylation activity toward the m6A modification on IFN-β mRNA, thereby modulating antiviral innate immune responses [75].

Collectively, the direct lactylation of RNA-modifying enzymes or m6A regulatory components provides a more straightforward molecular explanation for how lactate metabolic states govern m6A dynamics [74–76]. Although direct lactylation of RNA-modifying enzymes provides the most straightforward mechanistic link between lactate metabolism and m6A remodeling, current evidence remains limited to a small number of enzymes, lysine residues, and disease models. Therefore, these findings should not be generalized to all m6A regulators without further validation using site-specific mutants, rescue experiments, lactylomics, and m6A mapping.Importantly, the lactylation–m6A regulatory axes described above should be interpreted with caution regarding spatial and cellular heterogeneity. Within the tumor microenvironment, lactate concentrations are not uniform: hypoxic/necrotic regions exhibit markedly higher lactate levels than perivascular areas, leading to spatially heterogeneous histone lactylation patterns and, consequently, regionally variable m6A regulator expression. Moreover, different cell types—cancer cells, CAFs, TAMs, and lymphocytes—exhibit distinct glycolytic capacities and lactate sensitivities, resulting in cell-type-specific m6A–lactylation crosstalk. Thus, findings derived from single cell lines or bulk tissue analyses should not be overgeneralized as universal mechanisms.

### m6A-driven metabolic control of lactylation

In contrast to lactylation, which directly regulates m6A as an upstream signal, the influence of m6A on lactylation is primarily indirect and mediated through metabolic regulation. Recent studies suggest that m6A modification shapes intracellular lactate metabolism by modulating glycolytic flux, regulating the expression and stability of lactate-producing enzymes, and influencing metabolism-related signaling pathways (Fig. 3). These processes may alter the availability of lactylation substrates and the extent of protein lactylation.

Glycolytic flux directly determines lactate levels, and lactate serves as the essential metabolic source for protein lactylation. m6A modification regulates the RNA fate of various glucose metabolism genes, thereby altering glycolysis and lactate output. m6A writers or readers can enhance the stability and translation of glycolytic genes, such as those encoding aldolase and LDH, thereby increasing lactate production and providing more substrates for lactylation [1]. IGF2BP2, a recently identified m6A-binding protein, has been reported to enhance mRNA stability and translation. It promotes lactate accumulation by stabilizing ALDOA mRNA, thereby increasing ALDOA expression and facilitating histone H3K18 lactylation, ultimately accelerating liver fibrosis progression. Similarly, YTHDF1, a key m6A reader, directly binds to m6A-modified LDHA mRNA, enhancing its translation efficiency and enzymatic activity, which accelerates the conversion of pyruvate to lactate and elevates intracellular lactate levels [25] (Fig. 3). In addition, in a NAFLD model, LDHA-induced H3K18 lactylation promotes METTL3 expression, thereby enhancing m6A modification of SCD1 mRNA and stabilizing SCD1 through a YTHDF1-dependent mechanism, which contributes to lipid metabolic dysregulation [33].

In addition to directly regulating glycolytic genes, m6A modification is increasingly recognized as an important regulator of metabolic reprogramming. Emerging evidence suggests that metabolic flux, particularly glycolysis-derived lactate, can act as a signaling metabolite to drive histone lactylation and gene expression remodeling [1, 4]. Increasing evidence suggests that m6A regulates key metabolic signaling pathways, including PI3K/AKT, AMPK, and HIF-1α, all of which are closely associated with metabolic reprogramming. The PI3K/AKT pathway is a central regulator of cell proliferation and metabolism, promoting glucose uptake, glycolysis, and anabolic processes. m6A modification regulates the stability or translation of PI3K/AKT-related mRNAs (e.g., AKT and PDPK1), thereby enhancing pathway activity, increasing glycolytic flux, and promoting lactate production, which supports protein lactylation. Meanwhile, m6A also modulates AMPK signaling, a key energy sensor that is activated under energy stress conditions to inhibit anabolic metabolism and promote energy production. m6A influences the expression of AMPK-related genes (e.g., LKB1), thereby indirectly regulating AMPK activity. In the context of metabolic reprogramming, m6A-mediated regulation of AMPK may cooperate with PI3K/AKT signaling to form a complex metabolic regulatory network, allowing cells to dynamically adjust glycolysis and energy metabolism under varying conditions [40, 77]. Furthermore, under hypoxia, m6A regulates the HIF-1α signaling network [6]. HIF-1α is a master transcription factor in the hypoxia response and activates a wide range of glycolysis-related genes, such as GLUT1, HK2, and PFKFB3, thereby enhancing glycolysis-dependent survival [78]. m6A modification, by regulating demethylases such as ALKBH5, alters HIF-1α activity and promotes the high expression of these genes, thereby potentially increasing lactate production [6]. Therefore, m6A modification contributes to metabolic reprogramming by modulating key pathways such as PI3K/AKT, AMPK, and HIF-1α. Linking lactate production to protein lactylation supports a model of a sophisticated crosstalk network that collectively governs cellular metabolic states and functional phenotypes. In conclusion, m6A modification does not directly catalyze lactylation but acts as a “metabolic switch” to regulate lactate generation and homeostasis, building a critical indirect regulatory network between cellular metabolism and lactylation. This mechanism is particularly prominent in high-metabolic cells like tumor cells and activated fibroblasts, providing a theoretical basis for understanding cross-level interactions between m6A and lactylation (Fig. 3).

## m6A lactylation crosstalk in human diseases

In recent years, metabolic reprogramming has been recognized as a hallmark of various human diseases [79, 80]. It connects cellular metabolic states with epigenetic and epitranscriptomic regulation to participate in disease development [81]. m6A methylation, as the most abundant post-transcriptional modification, participates in gene expression regulation by modulating mRNA stability, splicing and translation efficiency. Lactate, a key metabolite of glycolysis, not only participates in energy metabolism but also functions as a signaling molecule by inducing protein lactylation, thereby regulating gene transcription in various pathological processes [1]. m6A and lactylation, as two metabolism-sensitive modifications, play key roles in metabolic reprogramming and disease [4]. Increasing evidence suggests that m6A and lactylation do not function independently; instead, they coordinately regulate transcription through a coupling mechanism between metabolic states and epigenetic regulation [82]. This interplay has been widely observed in multiple diseases, including cancer, metabolic and endocrine disorders, cardiovascular diseases, and inflammatory and immune-related conditions.

### Cancerous diseases

Tumor development is a complex process driven by multiple factors, among which metabolic reprogramming is considered a key hallmark. In particular, the “Warburg effect,” characterized by enhanced aerobic glycolysis, enables tumor cells to continuously produce large amounts of lactate even under normoxic conditions [79, 80]. In cancer, metabolic reprogramming drives excessive lactate accumulation, thereby inducing protein lactylation. Lactylation not only regulates gene transcription through chromatin remodeling but also directly targets m6A regulatory factors (writers, readers, and erasers), thereby altering m6A levels and reshaping downstream gene expression and mRNA fate. Simultaneously, m6A influences lactate metabolism and lactylation levels by regulating genes involved in glycolysis and lactate production, forming a reciprocal regulatory loop. This interaction establishes a multi-layered regulatory network within tumor systems [12, 17]. Therefore, in cancer, the interplay between m6A and protein lactylation not only constitutes a fundamental mechanism underlying metabolic reprogramming but also serves as a critical link connecting intrinsic metabolic alterations in tumor cells with remodeling of the tumor immune microenvironment. These mechanisms are widely involved in hematologic malignancies as well as tumors of the digestive, respiratory, urinary, and reproductive systems.

#### Hematological malignancies

Hematological malignancies, particularly acute myeloid leukemia (AML), represent one of the most well-characterized disease models for m6A-mediated tumor epitranscriptomic regulation. The development and progression of AML rely on the aberrant expansion of leukemic blasts and leukemia stem cells within the bone marrow microenvironment. Distinct from most solid tumors, AML develops within a highly dynamic hematopoietic niche, where bone marrow microenvironmental factors—including hypoxia, enhanced glycolysis, inflammatory signaling, stromal cell remodeling, and immunosuppression—collectively contribute to leukemic progression and therapeutic resistance [83–85]. Consequently, AML provides a critical disease context for exploring the potential interplay among metabolic reprogramming, m6A regulation, and lactate-associated epigenetic modifications.

To date, the functional significance of m6A regulation in AML has been substantially documented by numerous experimental studies. The m6A writer METTL3 is crucial for sustaining AML cell proliferation and an undifferentiated state, whereas its knockdown or pharmacological inhibition promotes myeloid differentiation, induces apoptosis, and impairs leukemia stem cell activity [86–88]. METTL14, a core component of the m6A methyltransferase complex, similarly exerts a pro-leukemic role in AML, where its silencing triggers terminal myeloid differentiation and suppresses AML cell survival and proliferation [89]. Furthermore, METTL16 has recently been revealed to promote leukemogenesis and the self-renewal of leukemia stem/initiating cells by modulating BCAT1/BCAT2 expression and branched-chain amino acid metabolism [90]. Together, these findings demonstrate that the m6A writing machinery is not only intricately involved in differentiation blockade and stemness maintenance in AML but may also influence the metabolic adaptation of leukemic cells by targeting metabolism-related transcripts.

m6A demethylases are similarly implicated in the progression of AML. FTO is aberrantly overexpressed in certain AML subtypes, where it promotes leukemic cell survival by reducing the m6A levels of specific transcripts, thereby stabilizing mRNAs associated with oncogene expression, differentiation block, or therapeutic resistance [91, 92]. In relapsed AML, elevated FTO expression correlates with chemoresistance, suggesting that FTO may contribute to AML relapse and treatment failure [93]. Another m6A demethylase, ALKBH5, has also been demonstrated to promote leukemogenesis and the self-renewal of leukemia stem cells, through mechanisms involving the ALKBH5/m6A/TACC3/MYC regulatory axis as well as the KDM4C-ALKBH5-AXL signaling axis [94, 95]. Together, these findings suggest that the functions of m6A writers and erasers in AML are not simply antagonistic, but are highly dependent on specific target transcripts, cellular states, and disease stages.

Beyond the intrinsic m6A regulation of leukemia cells, lactate accumulation and lactylation within the bone marrow microenvironment may provide a metabolic foundation for m6A–lactylation crosstalk. The AML bone marrow microenvironment is frequently characterized by hypoxia and enhanced glycolysis, leading to elevated lactate levels. Lactate functions not only as a metabolic product but also as a signaling molecule that modulates immune cell states. For instance, lactate promotes the accumulation of PD-1^+^regulatory T cells within high-tumor-burden AML bone marrow and participates in the formation of an immunosuppressive leukemic niche [96]. Additionally, recent studies have revealed that STAT5-mediated enhanced glycolysis and lactate accumulation promote histone lactylation. This process is associated with AML immunosuppression through the upregulation of PD-L1 expression [97]. Together, these insights suggest that the lactate–lactylation axis may be involved in AML bone marrow microenvironment remodeling and immune evasion.

However, in contrast to the mechanisms documented in certain solid tumors where lactylation directly regulates METTL3 or other RNA-modifying enzymes, a definitive m6A–lactylation feedback loop in AML currently lacks direct experimental evidence [62, 74, 76]. Consequently, the proposed “bone marrow niche–lactate–lactylation–m6A” regulatory axis in AML should currently be conceptualized as a speculative model based on existing metabolic, immunologic, and epigenetic evidence, rather than a definitively established mechanism [97, 98]. In one plausible scenario, hypoxia and enhanced glycolysis within the AML bone marrow microenvironment result in lactate accumulation, which may subsequently alter the transcriptional activity of genes encoding m6A regulators via histone lactylation; alternatively, non-histone lactylation might directly impinge upon the stability, subcellular localization, or catalytic activity of m6A-modulating enzymes. Conversely, m6A regulators may influence lactate production and lactylation dynamics within AML cells and their surrounding bone marrow microenvironment by targeting transcripts involved in glycolysis, lactate transport, mitochondrial metabolism, or immunometabolic adaptation. These hypothetical models warrant further validation through integrated approaches including lactylomics, m6A-seq, single-cell multi-omics, spatial metabolomics, and functional rescue assays [97, 98] (Table 2).

**Table 2:** Interaction between m6A methylation and lactylation in cancerous diseases

Disease type	Modified target molecule	Modification type	m6A reader/writer/eraser protein	Mechanism/regulatory axis	Biological outcome/cellular phenotype	Ref.
Hematological malignancy: AML (m6A component evidence)	Leukemia-associated transcripts regulated by METTL3, METTL14, METTL16, FTO, or ALKBH5 (e.g., BCAT1/2, TACC3, MYC, AXL, ITGA4)	m6A	METTL3; METTL14; METTL16; FTO; ALKBH5	m6A-dependent regulation of leukemic stemness, differentiation, metabolic adaptation, homing, and chemoresistance; component evidence supporting the proposed crosstalk	Leukemia stem-cell maintenance; differentiation blockade; metabolic adaptation; chemoresistance	[86–95, 99, 100]
Hematological malignancy: AML (lactate/lactylation component evidence)	PD-L1-related chromatin regions; immune-regulatory programs	Histone lactylation; lactate-associated epigenetic regulation	Not directly defined in this axis	Lactate/STAT5/histone lactylation/PD-L1 axis	Treg accumulation; immune suppression; bone-marrow niche remodeling	[96, 97]
Hematological malignancy: AML (proposed crosstalk)	m6A- and lactylation-related gene signatures	m6A; lactylation-related inference	Multiple m6A regulators; no single regulator directly validated for the complete axis	Proposed bone-marrow niche/lactate/lactylation/m6A coupling	Prognostic stratification; inferred metabolic–epigenetic remodeling	[98]
Digestive: Gastric cancer	H3K18; CCT2 mRNA	H3K18la; m6A	METTL3	Lactate/H3K18la/METTL3/CCT2 m6A axis	CD8 + T-cell dysfunction; tumor immune escape	[62]
Digestive: Gastric cancer	METTL16 lactylation; FDX1 mRNA	METTL16 lactylation; m6A	METTL16	METTL16-K229 lactylation/FDX1 m6A axis	Cuproptosis-related regulation; gastric cancer progression	[101]
Digestive: Colorectal cancer	PRMT5 K240; SLC7A11 mRNA	PRMT5 lactylation; m6A demethylation	ALKBH5	PRMT5-K240 lactylation/ALKBH5/SLC7A11 axis	Ferroptosis resistance; tumor progression	[68]
Digestive: Colorectal cancer	HDAC1; ferroptosis-related proteins	HDAC1 lactylation; m6A-related pathway association	m6A regulator not fully specified	HDAC1 lactylation-mediated ferroptosis-resistance pathway with m6A-network association	Ferroptosis resistance; therapy resistance	[69]
Digestive: Colorectal cancer	VIRMA; m6A-modified transcripts	VIRMA lactylation; m6A	VIRMA	CAF-derived circTAX1BP1/VIRMA lactylation/m6A axis	Tumor progression; microenvironmental remodeling	[70]
Digestive: Colorectal cancer	H3K18; IGF2BP2; NRF2 mRNA	H3K18la; m6A-dependent RNA stabilization	IGF2BP2	H3K18la/IGF2BP2/NRF2 m6A-stabilization axis	Ferroptosis resistance; tumor progression	[102]
Digestive: Colorectal cancer (m6A component evidence)	Kcnk6 mRNA	m6A	METTL3	METTL3/m6A/Kcnk6 inflammatory transcript-regulation axis; lactylation linkage remains indirect	Colon homeostasis; inflammation-associated carcinogenesis	[103]
Digestive: Liver cancer / HCC	IGF2BP3 K76; ferroptosis-related transcripts	IGF2BP3 lactylation; m6A-dependent RNA regulation	IGF2BP3	PARK7/IGF2BP3-K76 lactylation axis	Ferroptosis modulation; HAIC resistance	[71]
Digestive: Liver cancer / HCC	IGF2BP3; PCK2 and NRF2 mRNAs; serine-metabolism/SAM pathway	IGF2BP3 lactylation; m6A	IGF2BP3	Lactate/IGF2BP3 lactylation/PCK2–NRF2 stabilization/serine metabolism/SAM/m6A feedback axis	Metabolic reprogramming; lenvatinib resistance	[72]
Digestive: Liver cancer / HCC	H3K18; YTHDC1; lipid-metabolism-related mRNAs	H3K18la; m6A-dependent RNA regulation	YTHDC1	H3K18la/YTHDC1/lipid-metabolism remodeling axis	Lipid metabolic remodeling; HCC progression	[104]
Digestive: Pancreatic cancer / PDAC	METTL16 K269; CTCF mRNA	METTL16 lactylation; m6A	METTL16	Lactate sensing/METTL16-K269 lactylation/CTCF m6A-stabilization axis in tumor-associated Schwann cells	CD8 + T-cell dysfunction; immune evasion; resistance to PD-1 blockade	[76]
Digestive: Pancreatic cancer / PDAC	H3K18; HNRNPC; TRAF6 and ALDH1A3 mRNAs	H3K18la; m6A-dependent RNA stabilization	HNRNPC	H3K18la/HNRNPC/autophagy positive-feedback axis	Autophagy; metabolic reprogramming; gemcitabine resistance	[105]
Digestive: Pancreatic cancer / PDAC	IGF2BP2 promoter; CSF1 and MYC mRNAs	H3K18la; m6A-dependent RNA stabilization	IGF2BP2	FLG-AS1/CTCF/EP300/H3K18la/IGF2BP2 axis	Tumor proliferation; macrophage polarization; immune-microenvironment remodeling	[106]
Digestive: Esophageal SCC	H3K18; c-Myc mRNA	H3K18la; m6A	METTL3	AP001885.4/lactate/H3K18la/NF-kB/c-Myc transcription and METTL3-dependent m6A stabilization axis	Oncogenic mRNA stabilization; cell proliferation; tumor progression	[107]
Digestive: Esophageal SCC	PDIA3P1 transcript	Histone lactylation; m6A	WTAP	Histone lactylation/WTAP-mediated m6A/PDIA3P1 stabilization axis	Tumor progression	[108]
SCC-related inferred evidence	H3K18; METTL3/IGF2BP-related oncogenic transcripts	H3K18la; m6A	METTL3; IGF2BP family	Inferred SCC-associated lactylation–m6A axis; direct PSCC-specific experimental validation remains unavailable	Proliferation; invasion; immune evasion	[107–110]
Respiratory: NSCLC	H3K18; SFRP2 mRNA	H3K18la; m6A	YTHDF2	H3K18la/YTHDF2/SFRP2 m6A-recognition axis	Glycolysis; stemness; tumor progression	[111]
Respiratory: NSCLC	RBM15; m6A-modified tumor-associated transcripts	RBM15 lactylation; m6A	RBM15	RBM15 lactylation/stabilization/m6A-regulation axis	Tumor-cell growth and progression	[112]
Reproductive: TNBC / Breast cancer	METTL3; DNA-damage-repair-related transcripts	METTL3 lactylation/delactylation; m6A	METTL3; HDAC2	HDAC2/METTL3 delactylation/m6A-mediated DNA-damage-repair axis	DNA-damage repair; chemotherapy resistance; tumor survival	[26]
Urinary: Bladder cancer	YTHDF3; KDM6B mRNA	YTHDF3 lactylation; m6A-dependent RNA decay	YTHDF3	YTHDF3 lactylation/KDM6B m6A-decay axis	Impaired DNA-damage response; cisplatin resistance	[73]
Urinary: Bladder cancer	H3K18; IGFBP3 mRNA	H3K18la; m6A	RBM15	Hypoxia/H3K18la/RBM15/IGFBP3 m6A axis	Cisplatin resistance	[113]
Urinary: Bladder cancer	PFKFB4 mRNA	m6A; lactylation-linked glycolytic regulation	RBM15; IGF2BP3	RBM15/IGF2BP3/PFKFB4 m6A axis linked to glycolysis and lactate accumulation	PD-L1-related immune escape; tumor progression	[114]
Urinary: Renal cancer / RCC (bioinformatic signature)	m6A- and lactylation-related genes	Integrated transcriptomic signature	Multiple m6A regulators	m6A–lactylation modification-related prognostic and TME signature	Prognostic stratification; immune-microenvironment remodeling	[115]
Urinary: Renal cancer / RCC	YTHDC1; target mRNAs	YTHDC1 lactylation; m6A-dependent RNA stabilization	YTHDC1	YTHDC1 lactylation/phase separation/target-mRNA stabilization axis	Tumor adaptability; RCC progression	[116]
Ocular melanoma	H3K18; YTHDF2; PER1 and TP53 mRNAs	Histone lactylation; m6A-dependent RNA regulation	YTHDF2	Histone lactylation/YTHDF2/m6A-dependent transcript-regulation axis	Oncogenesis; tumor progression	[117]

Notes: Abbreviations: m6A, N6-methyladenosine; H3K18la, histone H3 lysine 18 lactylation; AML, acute myeloid leukemia; CAF, cancer-associated fibroblast; ESCC, esophageal squamous cell carcinoma; SCC, squamous cell carcinoma; PSCC, pharyngeal squamous cell carcinoma; HCC, hepatocellular carcinoma; PDAC, pancreatic ductal adenocarcinoma; NSCLC, non-small cell lung cancer; TNBC, triple-negative breast cancer; RCC, renal cell carcinoma; TME, tumor microenvironment. The slash “/” denotes a simplified functional or sequential relationship rather than a universally validated linear pathway. Rows explicitly labeled “component evidence,” “proposed,” or “inferred” indicate that the complete m6A–lactylation causal chain has not been directly demonstrated. Evidence classification is summarized separately in Table 4

From a translational perspective, AML offers a compelling opportunity to explore combinatorial strategies coupling m6A-targeted therapies with metabolic interventions. Small-molecule inhibitors of METTL3 have demonstrated potent anti-leukemic potential in preclinical models of AML, where targeting METTL3 suppresses leukemic growth, induces differentiation and apoptosis, and prolongs survival in vivo [88]. Furthermore, METTL3 has been shown to mediate AML homing, engraftment, and chemoresistance via ITGA4, suggesting that targeting METTL3 may help overcome microenvironment-dependent therapeutic resistance [100]. Targeting FTO or ALKBH5 may likewise impair leukemia stem cell maintenance or abrogate therapeutic tolerance in specific disease contexts [92–95]. Concurrently, the loss of METTL3 in bone marrow mesenchymal stem cells promotes AML chemoresistance, indicating that m6A regulation operates not only within leukemia cells themselves but also contributes to microenvironment-dependent resistance through bone marrow stromal cells [99]. Future investigations are required to further elucidate whether integrating m6A-targeted therapeutics with metabolic or immunometabolic interventions can more effectively eradicate leukemia stem cells and prevent disease recurrence. Importantly, because normal hematopoietic stem and progenitor cells similarly rely on m6A regulation and metabolic adaptation, therapeutic strategies must cautiously distinguish leukemia-specific vulnerabilities from the vital mechanisms required for maintaining normal hematopoiesis [83, 86, 89].

#### Digestive system tumors

In digestive system tumors, the crosstalk between m6A and protein lactylation is primarily reflected in lactate-driven transcriptional regulation and immune evasion. Enhanced glycolysis in tumor cells leads to lactate accumulation, which induces histone lactylation (e.g., H3K18la), thereby regulating the expression of m6A regulators and affecting the stability or translation of downstream target transcripts through m6A modification. In parallel, METTL16 lactylation directly regulates FDX1 mRNA through m6A modification and promotes cuproptosis. In gastric cancer, lactate-derived H3K18la upregulates METTL3, enhancing m6A-mediated CCT2 translation and impairing CD8⁺ T-cell survival, thereby contributing to tumor immune escape [62]. In addition, METTL16 lactylation at K229 has been reported to regulate FDX1 mRNA through m6A modification and promote cuproptosis in gastric cancer [101] (Table 2). However, the reverse m6A-driven glycolysis–lactate–lactylation feedback loop in gastric cancer still requires more direct validation. In colorectal cancer, lactate-induced histone and non-histone lactylation (e.g., H3K18la and HDAC1 lactylation) regulates m6A-related enzymes or readers, including ALKBH5, METTL3, VIRMA, and IGF2BP2 in different studies, thereby reshaping the m6A modification landscape. Conversely, m6A-mediated regulation of glycolytic or ferroptosis-related transcripts may further reshape tumor metabolism and lactylation-related stress responses. This interaction promotes tumor progression and therapy resistance by inhibiting ferroptosis and modulating the immune-inflammatory microenvironment [68–70, 102, 103] (Table 2). In liver cancer, lactate-driven histone lactylation upregulates m6A-related factors such as IGF2BP3, stabilizing metabolic transcripts in an m6A-dependent manner, thereby promoting metabolic reprogramming and drug resistance. Lactylation also modulates m6A-related readers and downstream metabolic or ferroptosis-related transcripts. Meanwhile, m6A enhances glycolysis and lactate production, forming a “m6A–metabolism–lactylation” positive feedback model associated with tumor progression [71, 72, 104] (Table 2). In pancreatic cancer, a multi-layered regulatory network has been proposed or observed in specific PDAC models between m6A modification and protein lactylation. In pancreatic ductal adenocarcinoma, enhanced glycolysis induces lactate accumulation and H3K18 lactylation, which upregulates the m6A regulator HNRNPC and stabilizes transcripts such as TRAF6 and ALDH1A3 in an m6A-dependent manner, thereby promoting autophagy and metabolic reprogramming [105]. Under diabetic or metabolically dysregulated PDAC conditions, lactate uptake through MCT1/MCT4 in tumor-associated Schwann cells has been reported to induce METTL16 K269 lactylation. This promotes METTL16-mediated m6A-dependent stabilization of CTCF and transcriptional activation of immunosuppressive ligands, thereby impairing CD8⁺ T cell function and contributing to tumor immune evasion and resistance to PD-1 blockade [76]. At the chromatin level, m6A and lactylation also cooperate; for example, CTCF recruits EP300 via lncRNAs to promote histone lactylation at the IGF2BP2 promoter, thereby activating IGF2BP2 and enhancing the stability of oncogenic transcripts such as CSF1 and MYC, collectively promoting tumor proliferation and immune microenvironment remodeling [106] (Table 2). Similarly, in esophageal squamous cell carcinoma, lncRNA AP001885.4 promotes lactate accumulation and histone lactylation, thereby activating NF-κB-dependent c-Myc transcription, while simultaneously upregulating METTL3-mediated m6A modification to enhance c-Myc mRNA stability. This suggests a coordinated regulatory axis at both transcriptional and post-transcriptional levels, driving tumor progression [107] (Table 2).

#### Respiratory and reproductive system tumors

In respiratory-related diseases, such as non-small cell lung cancer and pulmonary fibrosis, m6A modification and protein lactylation form a dynamic regulatory network through metabolic–epigenetic crosstalk. Enhanced glycolysis leads to lactate accumulation and histone lactylation (e.g., H3K18la), which activates pro-fibrotic and inflammatory gene transcription, whereas deacetylases such as HDACs and SIRT act antagonistically. At the post-transcriptional level, m6A maintains regulatory balance. m6A readers such as YTHDF1 promote the translation of delactylation-related factors or effectors (e.g., NREP), while METTL3 and FTO regulate glycolysis and lactate metabolism, thereby influencing lactylation levels. Lactylation upregulates YTHDF1 expression and enhances NREP translation, promoting TGF-β1 secretion and fibroblast activation, whereas delactylation partially reverses this pathway, collectively regulating pulmonary fibrosis progression [111, 112, 118] (Table 2).

In reproductive system cancers, such as triple-negative breast cancer, METTL3 enhances the stability and translation of glycolytic enzyme mRNAs (e.g., PKM2 and LDHA), thereby promoting lactate accumulation and inducing lactylation (e.g., H3K18la). This may further influence METTL3-related oncogenic pathways and is associated with therapy resistance [26] (Table 2).

#### Urinary system tumors and other related contexts

In urinary system diseases, the interplay between m6A and lactylation is widely observed in bladder cancer, renal cancer, and renal fibrosis. In bladder cancer, lactylation enhances the stability of YTHDF3 and regulates the degradation of m6A target mRNAs (e.g., KDM6B), thereby weakening DNA damage responses and promoting drug resistance. Meanwhile, histone lactylation enhances m6A modification of specific transcripts (e.g., IGFBP3) via RBM15, while m6A further promotes glycolysis and lactylation, contributing to immune evasion through PD-L1 expression [73, 113, 114] (Table 2). In renal fibrosis, lactylation-related changes may be associated with increased METTL3 and YTHDF1 signaling and pro-fibrotic activation [119] (Table 2). In renal cancer, lactylation and m6A-related signatures are associated with tumor adaptability and immune remodeling [115, 116] (Table 2).

In SCC-related contexts, particularly esophageal squamous cell carcinoma (ESCC), lactate accumulation and lactylation-associated epigenetic regulation may cooperate with m6A-dependent transcript stabilization and be associated with malignant progression. For example, lactate-induced histone lactylation can enhance oncogenic transcriptional programs, while m6A regulators such as METTL3 or WTAP may further stabilize tumor-promoting transcripts at the post-transcriptional level [107, 108]. These findings suggest that lactylation–m6A crosstalk may represent a broader regulatory mechanism in squamous cell carcinoma. However, direct experimental evidence specific to pharyngeal squamous cell carcinoma (PSCC) remains limited. Accordingly, this regulatory model should be regarded as SCC‑associated evidence rather than a mechanism definitively validated in PSCC [109] (Table 2).

It is important to note that within solid tumors, the m6A–lactylation crosstalk exhibits significant spatial and cellular heterogeneity. Tumor regions with severe hypoxia and high glycolytic activity—typically necrotic cores—show much higher lactate accumulation than well-vascularized peripheral areas, leading to regionally distinct lactylation patterns. Furthermore, different cell types within the tumor ecosystem respond divergently to lactate: cancer cells may upregulate histone lactylation to sustain proliferation, whereas CAFs and TAMs may undergo distinct lactylation-driven metabolic and functional reprogramming. Thus, the regulatory axes summarized in the following sections, though representative, are largely derived from specific models and require spatial or single-cell resolution validation. The therapeutic implications of these findings are further discussed in Sect.  6.1.

### Metabolic and endocrine diseases

Metabolic and endocrine diseases, including obesity, T2DM, MASLD, and lipid disorders, are often accompanied by metabolic remodeling, dysregulated glycolytic activity, and altered lactate metabolism. Lactate is not only a metabolic intermediate but can also induce protein and histone lactylation through lactate-derived lactyl groups, potentially involving lactyl-CoA-related intermediates, thereby directly participating in the regulation of gene expression [51, 56, 109]. Meanwhile, m6A RNA modification, as a typical metabolite-sensitive epitranscriptomic regulatory mechanism, depends on metabolic intermediates such as S-adenosylmethionine (SAM) and α-ketoglutarate (α-KG) for its installation and removal, enabling it to sense alterations in cellular metabolic states [12]. Recent studies have demonstrated that, in metabolic and endocrine diseases, the interplay between lactylation and m6A is primarily manifested in several aspects. In some disease models, lactate-driven lactylation has been reported to affect m6A-related regulators or readers, whereas in other contexts histone lactylation modulates the transcription of m6A enzymes. Conversely, m6A regulates glycolysis and lactate production, thereby influencing lactylation levels and forming a metabolic feedback loop that contributes to disease progression [33, 72, 110].

In metabolic dysfunction-associated steatotic liver disease (MASLD), a high-fat diet enhances glycolysis and promotes excessive lactate production. It has been reported that the m6A methyltransferase METTL3 is significantly upregulated in MASLD and regulates key glycolytic genes (e.g., GAPDH and TPI1) via m6A modification, thereby exacerbating metabolic dysregulation. Meanwhile, lactate induces histone lactylation (e.g., H3K18la), which is enriched at the METTL3 promoter region, thereby enhancing its transcription and further increasing m6A levels [34] (Table 3). Similarly, during the metabolic progression of liver fibrosis, the m6A reader IGF2BP2 is significantly upregulated and stabilizes the mRNA of glycolytic enzymes such as ALDOA, thereby enhancing glycolysis and lactate production. Lactate subsequently promotes histone lactylation and drives hepatic stellate cell (HSC) activation, thereby accelerating fibrosis progression [120] (Table 3). In addition, Under metabolic stress with enhanced glycolysis, accumulated lactate promotes lysine lactylation of the m6A reader IGF2BP3. Lactylated IGF2BP3 exhibits enhanced binding affinity for metabolic-related mRNAs (e.g., PCK2 and NRF2), thereby increasing their stability and expression. This process further elevates SAM levels via serine metabolism, ultimately enhancing m6A modification [72]. In metabolic inflammatory conditions, such as obesity-associated chronic inflammation, lactate accumulation may induce H3K18la and may be associated with altered METTL3 or FTO expression. This, in turn, alters m6A modification levels and regulates the stability of inflammation-related transcripts [1, 121] (Table 3).

**Table 3:** Interaction between m6A methylation and lactylation in other diseases

Disease type	Modified target molecule	Modification type	m6A reader/writer/eraser protein	Mechanism/regulatory axis	Biological outcome/cellular phenotype	Ref.
Metabolic: MASLD / NAFLD	H3K18; METTL3 promoter; SCD1, GAPDH, and TPI1 mRNAs	H3K18la; m6A	METTL3; YTHDF1	HFD/LDHA/lactate/H3K18la/METTL3/YTHDF1/SCD1 axis together with METTL3-dependent m6A regulation of glycolytic transcripts	Metabolic dysregulation; lipid accumulation; steatosis progression	[33, 34]
Metabolic: Liver fibrosis	IGF2BP2; ALDOA mRNA; H3K18	m6A-dependent RNA stabilization; H3K18la	IGF2BP2	IGF2BP2/m6A/ALDOA stabilization/glycolysis/lactate/H3K18la axis	Hepatic stellate-cell activation; glycolytic remodeling; fibrogenesis	[120]
Metabolic: Obesity / metabolic inflammation (inferred)	H3K18; METTL3; FTO; inflammatory transcripts	H3K18la; m6A-related association	METTL3; FTO	Proposed lactate/H3K18la/METTL3-or-FTO/m6A inflammatory-regulation network; disease-specific causal validation remains limited	Chronic metabolic inflammation; inflammatory transcript dysregulation	[1, 2, 9, 18, 20, 109, 110]
Cardiovascular: Heart failure	USP38 mRNA; HDAC3; histone substrates	m6A; histone lactylation	METTL7B	METTL7B/USP38 m6A degradation/HDAC3 degradation/histone lactylation axis	Improved cardiac remodeling; cardioprotection; heart-failure attenuation	[122]
Cardiovascular: Atherosclerosis	H3K9; SCD1-related endothelial transcripts	H3K9la; m6A-related transcript regulation	p300-related lactylation machinery; specific m6A regulator not fully defined	SLC22A6/lactate/H3K9la/SCD1 transcription with proposed m6A-dependent transcript regulation	Endothelial dysfunction; atherosclerotic progression	[123–125]
Cardiovascular: Myocardial injury / programmed cell death	Apoptosis-, ferroptosis-, and pyroptosis-related mRNAs and proteins	m6A; lactylation-related pathway overlap	Multiple m6A regulators	Proposed m6A–lactylation crosstalk in programmed cell-death networks	Myocardial injury; apoptosis; ferroptosis; pyroptosis	[124, 125]
Immune: Antiviral immunity	ALKBH5; IFN-beta mRNA	ALKBH5 lactylation; m6A demethylation	ALKBH5	Lactate/ALKBH5 lactylation/IFN-beta m6A demethylation axis	Increased IFN-beta expression; enhanced innate antiviral response	[75]
Immune: Pulmonary arterial hypertension	H3K18; METTL3/YTHDF2-related vascular and immune transcripts	H3K18la; m6A-related association	METTL3; YTHDF2	H3K18la-associated METTL3/YTHDF2 regulatory network	Vascular remodeling; cytokine production; immune-microenvironment imbalance	[126]
Immune: IBD / chronic inflammation (inferred)	Kcnk6 mRNA and inflammation-related transcripts	m6A; lactylation-related pathway overlap	METTL3; YTHDF2-related network	METTL3/m6A/Kcnk6 inflammatory regulation with proposed overlap with lactylation-associated immune pathways	Immune-cell infiltration; chronic inflammation; inflammation-associated carcinogenesis	[103]
Immune: ALKBH5-related inflammatory regulation	ALKBH5; RNF123; NLRP3-inflammasome-related transcripts	ALKBH5 lactylation; m6A-dependent regulation	ALKBH5	Lactylation-driven ALKBH5/RNF123/NLRP3-inflammasome axis	Reduced macrophage inflammasome activation in G6PT deficiency	[127]
Neurological: Intracerebral hemorrhage	H3K18; METTL3; LCN2 mRNA	H3K18la; m6A	METTL3	Lactate/H3K18la/METTL3/LCN2 m6A axis	Astrocyte activation; neuroinflammation	[128]
Neurological: Neuropathic pain	H3K18; IGF2BP2; CCT2 mRNA	H3K18la; m6A-dependent RNA stabilization	IGF2BP2	H3K18la/IGF2BP2/CCT2 m6A-stabilization axis	DRG sensory-neuron sensitization; pain-signal amplification	[129]
Oral: Periodontitis / periodontal ligament stem cells	H3K18; IGF2BP1; BMP2 mRNA	H3K18la; m6A-dependent RNA stabilization	IGF2BP1	H3K18la/IGF2BP1/BMP2 m6A-stabilization axis	Osteogenic differentiation; periodontal-tissue remodeling	[130]
Fibrotic: Pulmonary fibrosis	H3K18; NREP mRNA	H3K18la; m6A-dependent translation	YTHDF1	H3K18la/YTHDF1/m6A/NREP translation axis	TGF-beta1 secretion; fibroblast activation; fibrotic progression	[118]
Fibrotic: Renal fibrosis (m6A component evidence)	METTL3-regulated renal-fibrosis transcripts	m6A; lactylation-related inference	METTL3	METTL3-dependent renal-fibrosis regulation; direct lactylation–m6A coupling remains unvalidated	Fibroblast activation; pro-fibrotic signaling; cisplatin-related renal fibrosis	[119]
Immune context: Macrophage polarization	Arg1-associated chromatin regions; METTL3; JAK1 mRNA and other myeloid transcripts	Histone lactylation; METTL3 lactylation; m6A	METTL3; YTHDF-related readers	Tumor-derived lactate/histone lactylation/Arg1 transcription and METTL3 lactylation/JAK1 m6A axis	M2-like polarization; tumor-infiltrating myeloid-cell immunosuppression	[4, 58, 74]

Notes: Abbreviations: m6A, N6-methyladenosine; H3K18la, histone H3 lysine 18 lactylation; H3K9la, histone H3 lysine 9 lactylation; MASLD, metabolic dysfunction-associated steatotic liver disease; NAFLD, non-alcoholic fatty liver disease; PAH, pulmonary arterial hypertension; IBD, inflammatory bowel disease; DRG, dorsal root ganglion. The slash “/” denotes a simplified functional or sequential relationship. Rows labeled “component evidence” or “inferred” should not be interpreted as fully established causal pathways. Evidence classification is summarized separately in Table 4

Collectively, in metabolic and endocrine diseases, m6A modification and protein lactylation form a dynamic regulatory network through multi-layered interactions. This interplay plays a central role in diseases such as MASLD, liver fibrosis, and metabolic inflammation, providing important insights into disease mechanisms and potential therapeutic strategies.

### Cardiovascular diseases

Cardiovascular diseases (CVDs), including heart failure and atherosclerosis, are characterized by metabolic reprogramming and abnormal lactate accumulation. Recent studies suggest that lactate, beyond being a metabolic byproduct, may regulate gene expression by inducing histone and non-histone lactylation and has been implicated in inflammation, vascular dysfunction, and cardiac remodeling. Meanwhile, m6A RNA modification participates extensively in the pathological regulation of the cardiovascular system by modulating mRNA stability, translation efficiency, and splicing processes [124, 125] (Table 3). Emerging evidence suggests that the interplay between lactylation and m6A may exhibit disease-specific regulatory patterns in different types of CVDs. In cardiac hypertrophy and heart failure, the m6A-related protein METTL7B has been shown to regulate lactylation levels in an m6A-dependent manner. Specifically, METTL7B promotes m6A modification and degradation of USP38 mRNA, thereby reducing USP38 expression and enhancing the ubiquitination-mediated degradation of HDAC3. As HDAC3 is a key deacetylase, its reduction leads to increased histone lactylation levels, thereby improving cardiac remodeling and protecting cardiac function [122]. In atherosclerosis, enhanced glycolysis in endothelial cells results in lactate accumulation and significantly elevated H3K9 lactylation (H3K9la). This modification is associated with increased transcription of lipid metabolism genes (e.g., SCD1) via enzymes such as p300, thereby potentially contributing to endothelial dysfunction and atherosclerosis progression. Meanwhile, m6A modification may regulate the stability of these metabolic transcripts, suggesting a potential functional coupling between lactylation and m6A at the metabolic level [123]. Furthermore, in programmed cell death (PCD) processes associated with cardiovascular diseases, including apoptosis, ferroptosis, and pyroptosis, lactylation and m6A may participate in overlapping regulatory pathways. Lactylation may influence the expression of inflammation- and metabolism-related proteins, whereas m6A may affect the stability and translation of related mRNAs (Table 3). These two mechanisms may act synergistically or antagonistically within PCD signaling networks, thereby influencing myocardial injury and vascular pathology [125]. This cardioprotective example contrasts with the predominantly disease-promoting roles of lactylation described in many tumor and inflammatory settings. Therefore, histone lactylation should be interpreted as a context-dependent transcriptional mark rather than a uniformly pathogenic modification. Whether lactylation aggravates or alleviates disease likely depends on the activated gene program, the modified histone or non-histone substrate, and the cellular context in which lactate accumulation occurs. In summary, m6A modification and protein lactylation may form a complex multi-layered regulatory network involved in the development and progression of cardiovascular diseases, although the current evidence remains largely indirect or correlative.

### Immune and inflammatory diseases

Immune and inflammatory diseases, including infectious diseases, chronic inflammatory disorders, and immune imbalance-related conditions, are often accompanied by profound metabolic reprogramming. This metabolic shift leads to lactate accumulation and protein lactylation, which may regulate immune and inflammatory responses either by modifying m6A-related proteins or by altering the transcription of m6A regulators through histone lactylation. In antiviral immunity, lactylation can directly act on m6A regulatory proteins (Table 3). During DNA virus infection, lactate accumulation induces lactylation of the m6A demethylase ALKBH5, enhancing its binding to IFN-β mRNA and promoting its demethylation, thereby increasing IFN-β expression and strengthening innate immune responses [75]. This finding highlights a direct mechanism by which lactylation regulates immune gene expression via modification of m6A enzymes. In addition to direct regulation observed in specific contexts, lactylation may indirectly modulate the m6A system at the chromatin level. In inflammatory diseases such as pulmonary hypertension, lactate-induced histone lactylation (e.g., H3K18la) may be associated with increased expression of m6A writers such as METTL3 and readers such as YTHDF2, suggesting a potential link with immune cell function and cytokine production [126]. These observations suggest that lactylation may be linked to inflammatory microenvironment regulation through potential interactions between epigenetic regulation and RNA modification. In chronic inflammatory diseases, such as inflammatory bowel disease, lactylation-related genes have been reported to be significantly associated with immune cell infiltration and inflammatory pathways. These pathways exhibit substantial overlap with m6A regulatory networks, suggesting possible coordinated roles in immune and inflammatory regulation [103]. However, current studies mainly provide correlative evidence, and the precise molecular mechanisms underlying this interaction remain to be further elucidated. Moreover, under metabolically driven inflammatory conditions, lactylation may be associated with inflammatory responses involving m6A-related enzymes. For instance, lactylation has been shown to act on m6A demethylases such as ALKBH5, thereby influencing inflammatory signaling pathways and immune responses [127] (Table 3). Collectively, in immune and inflammatory diseases, current evidence suggests that m6A and protein lactylation may participate in overlapping immune signaling pathways, providing potential molecular insights into disease pathogenesis.

### Other diseases

Beyond the aforementioned conditions, the interplay between m6A and lactylation also plays important roles in other diseases. In acute brain injury, such as intracerebral hemorrhage, lactate accumulation induces H3K18 lactylation, which upregulates METTL3 expression and enhances m6A levels in these experimental models (Table 3). This, in turn, promotes the expression of inflammatory genes such as LCN2, correlates with astrocyte activation, and exacerbates neuroinflammation [128]. In neuropathic pain, H3K18 lactylation promotes the transcription of the m6A reader IGF2BP2, which stabilizes target mRNAs such as CCT2 in an m6A-dependent manner, thereby amplifying pain signaling [129] (Table 3). In periodontitis, lactate-induced H3K18la upregulates IGF2BP1 expression and enhances the stability of BMP2 mRNA via m6A modification, thereby promoting osteogenic differentiation [130] (Table 3). In melanoma, lactate-driven histone lactylation regulates immune-related gene transcription, while m6A modification cooperates at the post-transcriptional level. m6A readers such as YTHDF1 and IGF2BP enhance the stability of key immune and metabolic transcripts, whereas lactylation promotes their transcriptional activation at the chromatin level. Together, these mechanisms remodel the tumor immune microenvironment, promote immunosuppressive cytokine expression, and facilitate immune evasion. Meanwhile, m6A also enhances lactate production by regulating metabolic pathways, thereby indirectly promoting lactylation [117] (Table 3). In tumor-infiltrating bone marrow-derived cells, such as macrophages, tumor-derived lactate may induce histone lactylation, which is associated with the expression of immunosuppressive genes (e.g., Arg1) and M2-like polarization. Concurrently, m6A may regulate the stability and translation of selected immune-related transcripts (e.g., via YTHDF1/YTHDF2) at the post-transcriptional level (Table 3). In this context, lactylation may contribute to transcriptional activation, whereas m6A may help maintain selected transcript expression, together potentially shaping an immunosuppressive microenvironment.Furthermore, m6A may influence cellular responsiveness to lactate signaling by regulating metabolic pathways, thereby potentially affecting lactylation-related effects [74] (Table 3).

### Evidence classification and causality assessment of disease-related m6A–lactylation crosstalk

Current studies indicate that m6A RNA methylation and protein lactylation are functionally interconnected across multiple disease contexts, including solid tumors, hematological malignancies, metabolic diseases, cardiovascular diseases, inflammatory and immune-related diseases, fibrotic diseases, and other pathological contexts. However, the strength of evidence supporting these regulatory axes varies substantially among studies. Some mechanisms have been supported by in vitro perturbation experiments, animal models, and human tissue validation, whereas others are mainly based on bioinformatic prediction, transcriptomic correlation, or indirect inference.

To avoid overinterpreting available research evidence, this paper grades representative disease-related m6A-lactylation crosstalk axes from six dimensions: regulatory layer, representative regulatory axis, involved diseases/conditions, experimental/system type, evidence source, and nature of association. This classification helps distinguish directly validated mechanisms from correlative or inferential observations and provides a more balanced framework for interpreting the disease-specific findings summarized above. The evidence classification and causality assessment of representative disease-related m6A–lactylation crosstalk mechanisms are shown in Table 4. The evidence levels used in Tables 2 and 3 are further defined and summarized in Table 4, which distinguishes direct mechanistic experimental evidence from functional, correlative, bioinformatic, or literature-inferred evidence.

**Table 4:** Evidence classification of m6A–lactylation crosstalk across disease contexts

Regulatory layer	Representative regulatory axis	Disease/condition involved	Experimental/system type		Evidence source	Nature of association	Ref.
Histone lactylation-mediated transcriptional regulation of m6A regulators	H3K18la/METTL3/m6A axis	Gastric cancer; MASLD/NAFLD; sepsis-associated lung injury		In vitro cell models; animal models; disease tissue validation in selected studies	Experimental evidence	Indirect mechanistic regulation: lactate-induced histone lactylation regulates transcription of m6A regulators	[33, 61, 62]
Non-histone lactylation-mediated indirect regulation of the m6A network	HDAC1 or PRMT5 lactylation/ferroptosis-related m6A regulatory pathways	Colorectal cancer		In vitro cell models; functional perturbation assays	Experimental evidence	Functional or pathway-level association; lactylation affects m6A-related phenotypes indirectly	[68, 69]
Direct lactylation of m6A regulatory proteins	METTL3 lactylation/JAK1 m6A axis; METTL16-K229 or K269 lactylation/FDX1 or CTCF m6A axis; ALKBH5 lactylation/IFN-β m6A demethylation axis	Tumor-infiltrating myeloid cells; gastric cancer; pancreatic cancer/PDAC; antiviral immunity		In vitro cell models; animal models; site-specific mutant or rescue experiments in selected studies	Experimental evidence	Direct mechanistic evidence: lactylation modifies m6A writers/erasers and changes m6A-dependent RNA fate	[74–76, 101]
m6A-driven metabolic regulation of lactylation	IGF2BP2/ALDOA/lactate/H3K18la axis; METTL3/YTHDF1/SCD1 axis; IGF2BP3 lactylation/m6A metabolic feedback axis	Liver fibrosis; MASLD/NAFLD; HCC/metabolic stress-related models		In vitro cell models; animal models; metabolic and molecular validation	Experimental evidence	Indirect mechanistic regulation: m6A controls glycolysis or lactate production, thereby influencing lactylation	[33, 72, 120]
Integrated m6A–lactylation signatures or correlation-based prediction	m6A-lactylation-related gene signature; inferred SCC-related lactylation-m6A regulatory axis	RCC; PAH; SCC-related contexts; selected inflammatory or metabolic diseases		Human tissue datasets; TCGA or public database analysis; literature-based inference	Bioinformatic, correlative, or review-based evidence	Correlative or inferred association; causality remains insufficiently validated	[107–109, 115, 126]

**Notes**: Abbreviations: m6A, N6-methyladenosine; H3K18la, histone H3 lysine 18 lactylation; MASLD, metabolic dysfunction-associated steatotic liver disease; NAFLD, non-alcoholic fatty liver disease; PDAC, pancreatic ductal adenocarcinoma; HCC, hepatocellular carcinoma; RCC, renal cell carcinoma; PAH, pulmonary arterial hypertension; SCC, squamous cell carcinoma; IFN-β, interferon-β; TCGA, The Cancer Genome Atlas. The slash “/” denotes a simplified regulatory relationship rather than a strictly linear pathway. “Experimental/system type” indicates the main model used, including in vitro cells, in vivo animals, human tissues, or public datasets. “Experimental evidence” refers to wet-lab functional validation, whereas “Bioinformatic, correlative, or review-based evidence” refers to computational analysis, clinical association, or literature inference without complete functional validation. “Nature of association” distinguishes direct mechanistic evidence, indirect mechanistic regulation, pathway-level association, and correlative or inferred association

Based on this evidence hierarchy, the therapeutic implications of targeting the m6A–lactylation axis should be interpreted cautiously. Regulatory pathways supported by direct mechanistic and in vivo evidence may represent more promising translational targets, whereas axes based mainly on correlative or bioinformatic evidence require further experimental validation before therapeutic conclusions can be drawn.

### Context-dependent and bidirectional effects of m6A and lactylation

It should be emphasized that lactylation and m6A methylation are not unidirectional disease-promoting modifications. Instead, both modifications display strong context-dependent and bidirectional regulatory effects [10, 51, 59]. The biological consequences of lactylation or m6A modification depend on the modified substrate, target gene, reader protein, cell type, metabolic state, and disease stage. Therefore, apparently contradictory observations should not be interpreted as inconsistencies, but rather as evidence that metabolism-sensitive epigenetic and epitranscriptomic regulation is highly conditional.

For lactylation, most cancer-related studies emphasize its disease-promoting role. In lactate-enriched tumor microenvironments, histone lactylation can activate transcriptional programs associated with glycolysis, immune evasion, macrophage polarization, invasion, and therapy resistance. However, lactylation may also exert protective or adaptive effects in specific tissues. In cardiovascular disease, the METTL7B–USP38–HDAC3 axis provides a representative example [122]. METTL7B promotes m6A-dependent degradation of USP38 mRNA, which enhances ubiquitination-mediated degradation of HDAC3. Since HDAC3 acts as a deacetylase/delactylation-related suppressive factor, its reduction maintains histone lactylation marks such as H3K18la and H4K5la, thereby alleviating cardiac remodeling and improving cardiac function. This finding indicates that histone lactylation is not intrinsically pathogenic; rather, its effect is determined by the transcriptional programs activated in a specific cellular and pathological context.

A similar bidirectional pattern is observed for METTL3-mediated m6A regulation. In many cancer models, METTL3 enhances glycolytic reprogramming by increasing the m6A modification, stability, or translation of glycolysis-related transcripts and pro-growth signaling pathways, thereby supporting tumor proliferation and metabolic adaptation [10, 19, 32]. In contrast, in lipid metabolism or adipogenic contexts, METTL3-mediated m6A may exert inhibitory or stage-specific regulatory effects. For example, a similar context-dependent pattern is observed for METTL3-mediated m6A regulation. Notably, METTL3 exhibits divergent, or even opposite, regulatory effects across distinct metabolic pathways and cellular compartments. In macrophage-associated lipid metabolism, METTL3 deficiency in myeloid or macrophage-lineage cells can promote tumor progression by facilitating ISG15-mediated FASN upregulation and lipid metabolic remodeling, thereby supporting a pro-tumorigenic macrophage phenotype. Conversely, in glycolytic tumor-cell contexts, elevated METTL3 enhances glycolytic activity by stabilizing m6A-modified glycolysis-related transcripts or upstream regulators. For example, METTL3 activates the m6A–GLUT1–mTORC1 axis in colorectal cancer.

These findings suggest that the apparent paradox of METTL3 function largely depends on substrate specificity and cellular context. The functional outcome of METTL3 is dictated by the specific metabolic target transcripts being methylated and the metabolic state of the cell. Therefore, METTL3 should not be regarded as uniformly oncogenic or uniformly tumor-suppressive across all contexts; rather, its role is jointly determined by cellular lineage, disease context, metabolic pathway, and target mRNA landscape. This supports the view that METTL3-mediated m6A modification functions as a flexible rheostat in metabolic regulation rather than a rigid binary switch. The regulatory details and evidence levels corresponding to various diseases are summarized in Tables 2, 3 and 4 at the end of this article.

## Therapeutic targeting and translational challenges of the m6A–lactylation axis

A bidirectional regulatory relationship between m6A RNA methylation and protein lactylation has been observed in several disease models, providing potential therapeutic entry points for disorders driven by metabolic reprogramming, epigenetic dysregulation, and immune microenvironmental remodeling. Therapeutic strategies targeting this axis include small-molecule inhibitors of m6A writers and demethylases, HDAC-related interventions, inhibitors of lactate production or transport, and combination strategies. However, clinical translation remains challenging because this regulatory axis is highly dependent on cell type, disease stage, and metabolic context. Key unresolved issues include the incomplete definition of lactylation-modifying enzymes, difficulty in distinguishing causality from correlation, the pleiotropic biological effects of lactate, and the potential systemic toxicity of broad-spectrum interventions [1, 4, 7, 33, 61, 62, 74, 110].

### Current therapeutic strategies targeting m6A and lactylation pathways

Current interventions targeting the m6A–lactate–lactylation regulatory network fall into six categories. These include METTL3 inhibitors, FTO inhibitors, ALKBH5 inhibitors, histone deacetylase inhibitors, lactate metabolic pathway inhibitors, and combination strategies. These approaches act on multiple key nodes across the metabolic-epigenetic interaction network. Specifically, they modulate m6A writing, RNA demethylation, lactylation-related epigenetic regulation, and lactate synthesis or transmembrane transport. Compared with single-target drugs, combination regimens show greater promise. Tailoring these frameworks to molecular subtypes, cellular metabolic states, and disease microenvironments is crucial. This strategy may be more likely to improve the therapeutic relevance of targeting the m6A–lactylation axis [10, 12]. In acute myeloid leukemia (AML), METTL3 maintains leukemia cells in an undifferentiated state and promotes leukemogenesis. METTL3 is critically involved in myeloid malignant progression primarily through m6A-dependent translational regulation [86, 87]. STM2457, a selective small-molecule inhibitor of METTL3, provides a critical proof of concept for targeting m6A writers. Yankova et al. demonstrated that STM2457 suppresses METTL3 catalytic activity and reduces global m6A levels in AML cells [88]. This inhibition induces differentiation and apoptosis in leukemia cells and exerts potent anti-leukemic effects in vivo [88]. Additionally, METTL3 regulates bone marrow homing, engraftment, and chemoresistance in AML cells [99, 100]. This indicates that METTL3 inhibitors do more than alter intrinsic RNA metabolism; they may also disrupt the critical crosstalk between leukemia cells and the bone marrow microenvironment [99, 100].

From the perspective of the m6A–lactylation crosstalk axis, the therapeutic effects of METTL3 are highly context-dependent. On one hand, METTL3 regulates glycolysis-related transcripts to remodel cellular lactate production and metabolic adaptation. For instance, the m6A-dependent GLUT1–mTORC1 signaling axis drives metabolic reprogramming in colorectal cancer. Similarly, m6A-related mechanisms may modulate LDHA expression to enhance glycolytic flux in tumor cells [25, 36, 37]. On the other hand, lactate accumulation and histone lactylation reciprocally regulate METTL3 expression or function. For example, LDHA-mediated H3K18la promotes non-alcoholic fatty liver disease progression via the METTL3/YTHDF1/SCD1 axis. In sepsis-associated lung injury, H3K18la regulates METTL3 to alter the m6A modification of ACSL4. Furthermore, within the gastric cancer microenvironment, lactate-induced histone lactylation upregulates METTL3 to reshape CCT2 m6A modification and suppress CD8 + T cell survival [33, 61, 62]. Beyond histones, METTL3 itself can undergo non-histone lactylation. For instance, HDAC2-mediated delactylation of METTL3 promotes DNA damage repair. This mechanism directly contributes to chemoresistance in triple-negative breast cancer [26]. Consequently, METTL3 inhibitors may simultaneously disrupt RNA methylation, glycolytic pathways, and lactylation feedback loops. However, METTL3 also maintains vital physiological roles in normal hematopoiesis, immune homeostasis, and tissue repair. Systemic or long-term inhibition could therefore pose significant safety risks [16, 17, 86].

FTO was one of the first discovered m6A demethylases. By erasing m6A modifications on specific transcripts, it regulates RNA stability, alternative splicing, translation, and degradation. In AML, FTO acts as a pro-leukemic factor. It drives leukemia cell proliferation and blocks myeloid differentiation by demethylating m6A on key transcripts like MYC and CEBPA [91]. The oncometabolite R-2HG targets the FTO/M6A/MYC/CEBPA pathway to exert antitumor activity. This mechanism provides a solid foundation for targeting FTO therapeutically [92]. FTO is also linked to AML relapse and chemoresistance. Inhibiting FTO could therefore help overcome certain forms of treatment resistance [93]. Beyond R-2HG-related approaches, small-molecule FTO inhibitors such as FB23 and FB23-2 are being used to explore the therapeutic potential of FTO in AML. Developed by Huang et al., FB23 and FB23-2 directly bind to FTO to selectively inhibit its m6A demethylation activity. Among them, FB23-2 effectively suppresses AML cell proliferation and promotes differentiation and apoptosis [131]. Ultimately, FTO inhibitors function by restoring m6A modification levels on specific target transcripts. This destabilizes oncogenic RNAs, induces tumor cell differentiation, and enhances therapeutic sensitivity.

Within the m6A–lactylation crosstalk network, FTO inhibition can indirectly alter cellular glycolysis, lactate production, and lactylation levels. m6A modification is widely involved in tumor metabolic reprogramming. It spans multiple pathways, including glycolysis, glutamine metabolism, lipid metabolism, and mitochondrial function. Consequently, targeting FTO could reshape the cellular m6A transcriptome to further disrupt lactate metabolic homeostasis [19, 38, 40]. However, FTO function exhibits profound substrate specificity and disease heterogeneity. FTO regulates distinct target gene profiles across different malignancies or metabolic disorders. Its inhibitory effects vary depending on cell lineage, hypoxia levels, metabolic contexts, and the immune microenvironment. Therefore, future clinical applications of FTO inhibitors will require precise patient stratification. This process should be guided by molecular subtypes, FTO expression levels, m6A target transcript profiles, and lactate metabolic characteristics.

ALKBH5 is another core m6A demethylase. It primarily participates in RNA nuclear export, alternative splicing, transcript stability, and tumor microenvironment remodeling. Studies in AML show that ALKBH5 promotes tumorigenesis and maintains leukemia stem cell self-renewal. The KDM4C–ALKBH5–AXL signaling axis also serves as a critical pathway for leukemia stem cell survival [94, 95]. These findings highlight the therapeutic potential of targeting ALKBH5. From a target validation perspective, ALKBH5 is closely linked to immunotherapy response. Li et al. demonstrated that ALKBH5 knockout in tumor cells enhances anti‑PD‑1 efficacy and extends mouse survival [132]. This knockout modulates the tumor microenvironment by regulating lactate levels and immunosuppressive cell accumulation. Thus, ALKBH5 is more than just an m6A demethylase and acts as a crucial translational target linking m6A modification, lactate metabolism, and immunotherapy response. Currently, ALKBH5 inhibitors and chemical probes such as DDO‑2728 are being used in functional studies. Compared with METTL3 and FTO inhibitors, small‑molecule agents targeting ALKBH5 remain in the preclinical stage and lag behind in development. Further optimization is still required regarding their target selectivity, in vivo efficacy, pharmacokinetics, and safety profiles.

ALKBH5 plays a bidirectional regulatory role within the m6A–lactylation axis. On one hand, ALKBH5 regulates hypoxic adaptation, metabolic reprogramming, immune evasion, and treatment resistance via m6A modification. On the other hand, ALKBH5 itself is subject to regulation by lactylation. Studies show that ALKBH5 lactylation promotes host innate immune responses against DNA herpesvirus and monkeypox virus. This indicates that lactylation can directly alter the functional state of this m6A demethylase [75]. In diabetic retinopathy, lactylation-induced ALKBH5 targets RNF123 to promote retinal Müller cell activation. Conversely, in G6PT-deficiency-associated inflammation models, lactylation-driven ALKBH5 dampens NLRP3 inflammasome activation in macrophages [121, 127].

These findings demonstrate that ALKBH5 serves as both a potential therapeutic target and a functional responder to cellular lactate fluctuations. Crucially, ALKBH5 can either drive pathological damage or exert immunomodulatory and tissue-protective effects depending on the disease context. Therefore, before developing ALKBH5 inhibitors, researchers must clearly define its functional direction in specific diseases and cell types.

Histone deacetylase (HDAC) inhibitors were among the earliest epigenetic drugs to enter clinical use. Representative agents include vorinostat, romidepsin, belinostat, and panobinostat. Traditionally, these inhibitors were thought to exert antitumor effects primarily by elevating histone acetylation, which relaxes chromatin conformation and reprograms the transcriptional profile. However, recent advances in lactylation research reveal a more complex relationship. In vitro enzymatic and cellular assays suggest that HDAC1–3 and certain sirtuins possess delactylation activity. Still, their substrate scope, site selectivity, and cell-type dependence require further definition [26, 56, 59, 69]. Meanwhile, HDAC proteins themselves can undergo lactylation, altering their own functions. For example, HDAC2-mediated delactylation of METTL3 promotes DNA damage repair and contributes to chemoresistance in triple-negative breast cancer [26, 69]. Conversely, HDAC1 lactylation is linked to ferroptosis resistance in colorectal cancer [26, 69].

Therefore, within the context of lactylation regulation, HDAC inhibitors cannot simply be classified as “anti-lactylation drugs.” Instead, they likely affect both deacetylation and delactylation. This dual action reshapes the cellular balance between these two modifications. As a result, it triggers bidirectional transcriptional effects that depend heavily on specific gene loci, cell types, and metabolic environments.

Their potential therapeutic value is reflected mainly in three areas. First, they alter chromatin accessibility, which regulates the transcription of m6A-related enzymes or reader proteins. Second, they reshape the histone lactylation landscape, thereby influencing the expression of immune- and metabolism-related genes. Third, by modulating non-histone lactylation, they impact the stability and functional activity of m6A regulators, metabolic enzymes, and signaling molecules. Because l actylation and acetylation share lysine modification sites, they may compete for the same acyl-CoA metabolic pool. Future research will need to leverage site-specific mass spectrometry, global histone profiling, and functional rescue assays. These approaches will be crucial to clarify the precise effects of HDAC inhibitors on the m6A–lactylation axis.

The lactate metabolic pathway is a central hub linking glycolysis, tumor microenvironment acidification, GPR81 receptor signaling, and protein lactylation. Lactate is mainly generated through the reduction of pyruvate by LDHA. Its transmembrane transport is primarily mediated by the monocarboxylate transporters MCT1 and MCT4. Targeting LDHA or the MCT family may reduce lactate accumulation and alter intracellular and extracellular pH status. It may also limit the substrate supply for lactylation and relieve lactate-induced immunosuppression in the microenvironment [42, 43, 49, 58]. Previous studies have shown that LDHA-dependent lactate accumulation and histone lactylation are involved in the pathological progression of non-alcoholic fatty liver disease, sepsis-associated lung injury, and various malignancies [33, 61, 62]. In AML, lactate can promote the accumulation of PD-1⁺ regulatory T cells in the bone marrow microenvironment with high tumor burden. STAT5 can also upregulate PD-L1 expression through histone lactylation and drive immune suppression. These findings suggest that inhibitors targeting lactate metabolism may have the potential to enhance combined immunotherapy [96, 97].

Key strategies for targeting the lactate pathway include inhibiting MCT1-mediated lactate transport and blocking LDH/LDHA-mediated lactate production. The MCT1 inhibitor AZD3965 has completed a Phase I dose-escalation study in patients with advanced tumors. This trial demonstrates that MCT1-mediated transport is clinically actionable [133]. Recent studies show that pharmacological LDH inhibition redirects intratumoral glucose uptake. This shift improves T-cell metabolic availability and boosts anti-tumor immunity in solid tumor models [134]. Additionally, combination studies with GNE-140 reveal a synergistic anti-tumor effect. Simultaneously restricting LDH-mediated lactate production and mitochondrial oxidative phosphorylation provides an experimental basis to overcome the metabolic compensation caused by LDH inhibition [135]. Mechanistically, MCT inhibitors can block lactate exchange among tumor cells, stromal cells, and immune cells. LDHA inhibitors can reduce lactate synthesis at its source. This may decrease the substrate supply for lactylation. However, lactate-targeted intervention carries a clear risk of metabolic compensation. Tumor cells may maintain metabolic survival by upregulating oxidative phosphorylation, glutamine metabolism, fatty acid oxidation, or alternative lactate transport pathways [23, 79, 80]. In addition, lactate is not only a pathological signaling molecule. It is also an important energy substrate and a mediator of intercellular energy shuttling. Long-term or broad inhibition of lactate metabolism may therefore disturb energy homeostasis in normal tissues [82]. Thus, LDHA and MCT inhibitors may be more suitable for disease subtypes with clear lactate- or lactylation-dependent features. They may also be combined with m6A-targeted agents, immune checkpoint inhibitors, or mitochondrial metabolism modulators.

Combination therapy is one of the most translationally valuable directions for targeting the m6A–lactate–lactylation network. This regulatory axis is a dynamic network. It tightly links RNA epigenetic modification, glycolytic metabolism, protein post-translational modification, and the immune microenvironment. Blocking a single node is usually not sufficient to fully suppress disease progression. The first strategy is to combine m6A regulator inhibitors with lactate metabolism inhibitors. For example, METTL3, FTO, or ALKBH5 inhibitors may be combined with LDHA or MCT inhibitors. This approach may interfere with RNA epigenetic reprogramming and the lactate–lactylation substrate supply at the same time. The second strategy is to combine lactate pathway inhibitors with immune checkpoint inhibitors. This may relieve lactate-mediated T cell dysfunction, Treg accumulation, myeloid cell-mediated immunosuppression, and PD-L1 upregulation [49, 58, 74, 96, 97]. The third strategy is to combine HDAC inhibitors with m6A-targeted small molecules. This may synergistically reshape the multilayered epigenetic network involving acetylation, lactylation, and m6A modification [26, 69]. The fourth strategy is to combine LDHA or MCT inhibitors with inhibitors of mitochondrial metabolism or glutamine metabolism. This may reduce compensatory metabolic bypass in tumor cells [47, 79, 80]. The key to rational combination therapy is not the simple addition of drugs. Rather, it is network-based synergistic intervention. Such intervention should focus on key nodes that have been validated to play causal roles in disease. However, whether these agents directly modulate the m6A–lactylation feedback loop remains to be fully validated.

### Translational challenges and unresolved controversies

Although the above targeted agents and therapeutic strategies have shown potential for modulating the m6A–lactylation axis in preclinical models, their clinical translation still faces four fundamental bottlenecks. These issues also represent the major controversies in this field. First, the identity and specificity of lactylation writers and erasers remain incompletely defined. Second, it is still difficult to strictly distinguish causal relationships among lactate accumulation, protein lactylation, altered m6A modification, and disease phenotypes. Third, the multiple biological effects of lactate are often intertwined. This may lead to imprecise mechanistic attribution. Fourth, both m6A modification and lactylation are highly context dependent. Broad-spectrum intervention may therefore carry a high risk of toxicity. These unresolved issues not only affect the interpretation of basic mechanisms. They also directly determine target selection, drug combination design, and patient stratification strategies.

#### Controversies over lactylation-specific regulatory enzymes

Since histone lactylation was systematically reported, acyltransferases such as p300/CBP have been considered to have lactylation-writing activity. HBO1/KAT7 has also been reported to participate in lactylation deposition at specific genomic loci [3, 56]. At the same time, HDAC1–3 and several deacetylation-related proteins have been shown to possess delactylation activity [26, 56, 59, 69]. These findings suggest that lactylation is not merely a non-enzymatic modification caused by lactate accumulation. At least to some extent, it is under enzymatic regulation. However, these enzymes cannot yet be simply defined as specific “lactylation writers” or “delactylases”. The classical physiological functions of most candidate enzymes are related to acetylation or broad lysine acylation. Their catalytic activity toward lactylation may reflect catalytic multifunctionality. It may not indicate specific recognition of lactyl-CoA as a substrate.The root of this controversy lies in the overlap between lactylation and acetylation. Both modifications occur on lysine residues. They may also be jointly affected by changes in the metabolic pools of lactyl-CoA, acetyl-CoA, and other acyl-CoAs. Therefore, an observed change in lactylation after manipulating a given enzyme does not directly prove that this enzyme specifically catalyzes lactylation. The effect may also result indirectly from broad acyltransferase activity, substrate competition, altered chromatin status, or changes in the ratio of different acyl-CoAs. Future studies should integrate in vitro reconstituted enzymatic assays, isotope-based lactate tracing, site-specific mass spectrometry, catalytically inactive mutants, substrate competition assays, and histone and non-histone lactylome profiling. These approaches are needed to define the direct catalytic capacity and substrate selectivity of candidate enzymes toward lactylation. Only when this issue is clarified at the enzymatic level can therapeutic strategies targeting lactylation-modifying enzymes have a solid mechanistic basis.

#### The causal relationship in the m6A–lactylation feedback loop remains difficult to verify

In various disease models, enhanced glycolysis, lactate accumulation, increased protein lactylation, m6A remodeling, and aggravated disease phenotypes often occur at the same time [74]. Typical bidirectional loops include LDHA-mediated H3K18 lactylation regulating METTL3-related m6A signaling [33]. Lactate-induced histone lactylation can also upregulate METTL3 and reshape immunosuppression-related transcripts [62]. In addition, lactylation-driven METTL3 can regulate the immunosuppressive function of tumor-infiltrating myeloid cells. Moreover, several m6A-related enzymes or reader proteins, including METTL16, ALKBH5, VIRMA, IGF2BP3, YTHDF3, and YTHDC1, have been reported to be regulated by lactylation-related mechanisms [70–75]. These changes further affect cell death, innate immunity, chemoresistance, metabolic reprogramming, and tumor progression [73, 74, 101, 116]. These studies suggest a close relationship between m6A modification and lactylation. However, synchronous changes do not necessarily indicate causal regulation. Bidirectional positive feedback loops further increase the difficulty of defining causality. m6A modification can promote lactate production by regulating glycolysis, HIF-1α, PI3K/AKT, AMPK, and related pathways. In turn, lactate and lactylation can regulate m6A-related enzymes through chromatin remodeling or direct protein modification [6, 7, 33, 61, 62, 77, 78]. Therefore, it remains unclear whether lactylation acts as a disease-driving factor or simply as a concomitant consequence of metabolic reprogramming.This question requires stricter experimental evidence. Lactate treatment, LDHA overexpression, increased pan-Kla levels, or altered expression of m6A regulators alone is not sufficient to prove causality. Future studies should use site-specific lysine lactylation mutants, catalytically inactive mutants of modifying enzymes, isotope-based time-course multi-omics, m6A sequencing, lactylome proteomics, and functional rescue assays. Only when mutation of a single lactylation site markedly abolishes m6A remodeling or disease phenotypes, and reintroduction of the wild-type protein restores the phenotype, can this pathway be more strongly defined as a lactylation-dependent causal regulatory mechanism.

#### Disentangling lactylation from the multiple biological effects of lactate remains challenging

Lactate is a multifunctional metabolic molecule. It can act as an energy substrate, an extracellular signaling ligand, a pH-regulating factor, and a substrate for lactylation [5, 42, 82]. On the one hand, lactate can be converted into lactyl-CoA. It can then modify lysine residues on histone and non-histone proteins. This process may alter chromatin accessibility, transcription factor activity, RNA-binding protein stability, and enzymatic activity [4, 51, 53, 55, 59]. On the other hand, lactate accumulation can also act through lactylation-independent mechanisms. It can acidify the microenvironment and directly affect tumor invasion, immune cell function, and drug sensitivity. The LDHA-catalyzed reaction can also change the NAD⁺/NADH redox balance. This further regulates glycolytic flux, mitochondrial function, and reactive oxygen species homeostasis. In addition, lactate can bind to the membrane receptor GPR81/HCAR1. Through downstream signals such as cAMP, this pathway regulates inflammatory responses, immune escape, and tumor cell proliferation and invasion [42–48, 79]. Therefore, it is not rigorous to attribute all phenotypes induced by lactate treatment or lactate accumulation to protein lactylation. To prove that a phenotype is mediated by protein lactylation, three major confounding factors must be excluded. These include acidosis, redox imbalance, and GPR81 receptor signaling. At the experimental level, several approaches may be used. These include pH-matched sodium lactate and lactic acid controls, buffered culture systems, GPR81 knockdown or receptor antagonism, NAD⁺/NADH and ROS detection, cellular metabolic flux analysis, LDHA or MCT intervention, lysine lactylation site mutation, and functional rescue assays. Only when blockade of lactylation itself markedly weakens the disease phenotype, while the major contributions of pH, redox status, and GPR81 signaling are excluded, can this phenotype be more reliably defined as a lactylation-dependent effect.This issue is particularly important in studies of tumor immunity, inflammation, fibrosis, and metabolic diseases. In these disease contexts, lactate accumulation, acidification, redox disturbance, immune suppression, and lactylation often occur simultaneously [49, 50, 58, 120].

#### Context-dependent bidirectional effects increase the difficulty of clinical translation

Neither m6A modification nor lactylation acts as a unidirectional pro-injury signal. Their functions vary across cell types, tissue states, disease stages, and metabolic contexts. In some settings, they may even exert opposite effects. For example, METTL3 can promote glycolysis and tumor progression in colorectal cancer through the GLUT1–mTORC1 axis [37]. However, in the context of macrophage lipid metabolism, METTL3 deficiency can promote glioma progression through the ISG15–FASN axis. This suggests that the same m6A writer may produce completely different functional consequences in different cell lineages [35]. Similarly, lactylation can promote immunosuppression and therapeutic tolerance in many tumors. However, it may also have adaptive or protective roles in immune regulation, tissue repair, and cardiovascular diseases. For instance, METTL7B-induced histone lactylation can prevent heart failure by improving myocardial remodeling. In contrast, SLC22A6-dependent H3K9 lactylation can aggravate endothelial dysfunction and promote atherosclerosis [122, 123]. This context dependence has important translational implications. Broad inhibition of METTL3, FTO, ALKBH5, HDACs, LDHA, or the MCT family may bring therapeutic benefits in certain diseased tissues. However, it may also disrupt metabolic homeostasis, immune function, hematopoiesis, or tissue repair in normal tissues. ALKBH5 is a typical example. In AML, ALKBH5 can maintain leukemia stem cells and drive tumorigenesis [94, 95]. However, lactylated ALKBH5 may also exert distinct functions in innate immunity and inflammatory regulation [75, 127]. Therefore, future therapies should not simply aim to “inhibit a modification” or “reduce a metabolite”. Instead, it is necessary to determine whether a given target acts as a pathogenic factor or a protective factor in a specific disease context. A more desirable strategy would integrate patient molecular subtyping, cell-specific markers, metabolic status, lactylome profiles, and m6A target transcript maps. These approaches may support the development of tissue-specific, microenvironment-responsive, or temporally controlled delivery systems. This may enable more precise targeted intervention.

### Future research directions

Future studies on the m6A-lactylation axis must shift from descriptive, single-molecule observations to systematic causal validation. Research needs to map the precise regulatory directions and causal relationships among lactate accumulation, protein lactylation, m6A remodeling, and disease phenotypes. To achieve this, future work should integrate site-specific lactylation mutations with stable isotope-labeled lactate tracing. It is also critical to combine time-course multi-omics and lactylation proteomics with m6A sequencing and functional rescue assays. For translational drug development, researchers must avoid broad-spectrum inhibition. This approach risks disrupting physiologically protective m6A or lactylation events. Instead, patients should be strictly stratified based on disease subtype, cell origin, metabolic state, and immune microenvironment profiles. Additionally, advanced delivery technologies are essential to enhance on-target efficacy and limit systemic toxicity. Key development areas include tissue- or cell-specific drug carriers and disease microenvironment-responsive delivery vehicles. Combination therapies represent the most promising strategy for clinical translation. This approach pairs m6A-targeted small molecules with lactate metabolism inhibitors or HDAC inhibitors. Other effective combinations include immune checkpoint inhibitors, as well as mitochondrial or glutamine metabolism regulators. Ultimately, transforming the m6A-lactylation axis from a bench concept into a clinically actionable intervention depends on a solid foundation. It requires rigorous pathway causality, standardized patient stratification, and precision drug delivery systems.

## Conclusion and perspectives

m6A RNA methylation and protein lactylation represent two metabolically responsive regulatory modalities that bridge cellular metabolic states with post-transcriptional regulation and protein post-translational modification. Under metabolic reprogramming, lactate accumulation may influence m6A-associated machinery through histone or non-histone lactylation, whereas m6A modification can reshape glycolysis and lactate metabolism by regulating metabolism-related transcripts, thereby indirectly affecting cellular lactylation status. Together, the m6A–lactylation axis provides a conceptual framework for understanding how metabolic cues are translated into layered epitranscriptomic and epiproteomic regulation in human diseases.

Despite growing evidence, this field remains at an early stage. Key unresolved issues include the causal relationship between lactate accumulation, lactylation, m6A remodeling, and disease phenotypes; the spatial and cellular heterogeneity of this crosstalk; the incomplete definition of lactylation writers, erasers, and readers; and the safety challenges associated with therapeutic targeting of metabolism-sensitive regulatory pathways. Future studies should integrate single-cell sequencing, spatial transcriptomics, spatial metabolomics, lactylome profiling, m6A-seq, site-specific lactylation mutants, and functional rescue assays to establish causal mechanisms and define disease- and cell-type-specific regulatory networks. Such efforts will be essential for translating the m6A–lactylation axis from a conceptual framework into a therapeutically actionable strategy.

## Data Availability

Not applicable.

## Abbreviations

ALKBH1: AlkB homolog 1
ALKBH5: AlkB homolog 5, RNA demethylase
α-KG: Alpha-ketoglutarate
AML: Acute myeloid leukemia
AMPK: AMP-activated protein kinase
ATP: Adenosine triphosphate
CAF: Cancer-associated fibroblast
cAMP: Cyclic adenosine monophosphate
CD8: Cluster of differentiation 8
CoA: Coenzyme A
CVD: Cardiovascular disease
DNA: Deoxyribonucleic acid
DRG: Dorsal root ganglion
ECM: Extracellular matrix
EMT: Epithelial–mesenchymal transition
ESCC: Esophageal squamous cell carcinoma
FTO: Fat mass and obesity-associated protein
GPR81/HCAR1: G protein-coupled receptor 81/hydroxycarboxylic acid receptor 1
H3K18la: Histone H3 lysine 18 lactylation
H3K9la: Histone H3 lysine 9 lactylation
H4K5la: Histone H4 lysine 5 lactylation
HBO1/KAT7: Histone acetyltransferase binding to ORC1/lysine acetyltransferase 7
HCC: Hepatocellular carcinoma
HDAC: Histone deacetylase
HIF-1α: Hypoxia-inducible factor 1 alpha
HNRNPA2B1: Heterogeneous nuclear ribonucleoprotein A2/B1
HNRNPC: Heterogeneous nuclear ribonucleoprotein C
HSC: Hepatic stellate cell
IBD: Inflammatory bowel disease
ICH: Intracerebral hemorrhage
IFN-β: Interferon beta
IGF2BP1/2/3: Insulin-like growth factor 2 mRNA-binding protein 1/2/3
JAK: Janus kinase
KAT: Lysine acetyltransferase
Kla: Lysine lactylation
LDH: Lactate dehydrogenase
LDHA/LDHB: Lactate dehydrogenase A/B
lncRNA: Long non-coding RNA
LPS: Lipopolysaccharide
m6A: N6-methyladenosine
m6A-seq: N6-methyladenosine sequencing
MASLD: Metabolic dysfunction-associated steatotic liver disease
MCE: Mitotic clonal expansion
MCT1/MCT4: Monocarboxylate transporter 1/4
METTL3/14/16/7B: Methyltransferase-like protein 3/14/16/7B
mRNA: Messenger RNA
mTORC1: Mechanistic target of rapamycin complex 1
NAD+/NADH: Oxidized/reduced nicotinamide adenine dinucleotide
NADP+: Nicotinamide adenine dinucleotide phosphate
NAFLD: Non-alcoholic fatty liver disease
NF-κB: Nuclear factor kappa B
NSCLC: Non-small cell lung cancer
OXPHOS: Oxidative phosphorylation
p300/CBP: E1A-binding protein p300/CREB-binding protein
PAH: Pulmonary arterial hypertension
PCD: Programmed cell death
PD-1: Programmed cell death protein 1
PDAC: Pancreatic ductal adenocarcinoma
PD-L1: Programmed death-ligand 1
PGC-1α: Peroxisome proliferator-activated receptor gamma coactivator 1 alpha
PI3K/AKT: Phosphoinositide 3-kinase/protein kinase B
PRISMA: Preferred Reporting Items for Systematic Reviews and Meta-Analyses
PSCC: Pharyngeal squamous cell carcinoma
PTM: Post-translational modification
R-2HG: R-2-hydroxyglutarate
RBM15: RNA-binding motif protein 15
RCC: Renal cell carcinoma
RNA: Ribonucleic acid
ROS: Reactive oxygen species
rRNA: Ribosomal RNA
SAM: S-adenosylmethionine
SCC: Squamous cell carcinoma
SIRT: Sirtuin
STAT: Signal transducer and activator of transcription
T2DM: Type 2 diabetes mellitus
TAM: Tumor-associated macrophage
TCA: Tricarboxylic acid cycle
TCGA: The Cancer Genome Atlas
TGF-β1: Transforming growth factor beta 1
TME: Tumor microenvironment
TNBC: Triple-negative breast cancer
Treg: Regulatory T cell
VIRMA/KIAA1429: Vir-like m6A methyltransferase-associated protein/KIAA1429
WTAP: Wilms tumor 1-associated protein
YAP: Yes-associated protein
YTHDC1/YTHDC2: YTH domain-containing protein 1/2
YTHDF1/2/3: YTH N6-methyladenosine RNA-binding protein 1/2/3
